# Solid state characterization and theoretical study of non-linear optical properties of a Fluoro-*N*-Acylhydrazide derivative

**DOI:** 10.1371/journal.pone.0175859

**Published:** 2017-04-24

**Authors:** Rosemberg F. N. Rodrigues, Leonardo R. Almeida, Florisberto G. dos Santos, Paulo S. Carvalho, Wanderson C. de Souza, Kleber S. Moreira, Gilberto L. B. de Aquino, Clodoaldo Valverde, Hamilton B. Napolitano, Basílio Baseia

**Affiliations:** 1 Campus de Ciências Exatas e Tecnológicas, Universidade Estadual de Goiás, Anápolis, Goiás, Brazil; 2 Universidade Paulista (UNIP), Goiânia, Goiás, Brazil; 3 Instituto de Física de São Carlos, Universidade de São Paulo, São Carlos, São Paulo, Brazil; 4 Instituto de Física, Universidade Federal de Goiás, Goiânia, Goiás, Brazil; 5 Departamento de Física, Universidade Federal da Paraíba, João Pessoa, Paraíba, Brazil; Oregon State University, UNITED STATES

## Abstract

In this work we determine the linear and non-linear optical properties of a Fluoro-*N*-Acylhydrazide derivative (FBHZ), using a combined supermolecule approach and an iterative scheme of electrostatic polarization, where the atoms of neighbouring molecules are represented by point charges. Our results for non-linear optics (NLO) are comparable to those found experimentally, suggesting that FBHZ constitutes an attractive object for future studies and for use as an interesting material for third-order NLO applications. The dynamic electrical properties of FBHZ in different solvent media are reported. Its molecular properties are closely related to supramolecular features; accordingly, we analysed all its crystal structure properties via intermolecular interactions in the solid state, using X-ray crystallography data and Hirshfeld surface (HS), including thermogravimetric analysis (TGA), differential scanning calorimetry (DSC) and hot-stage microscopy (HSM), where the results reveal crystal stability in respect to temperature variation.

## Introduction

**Fig 1:**
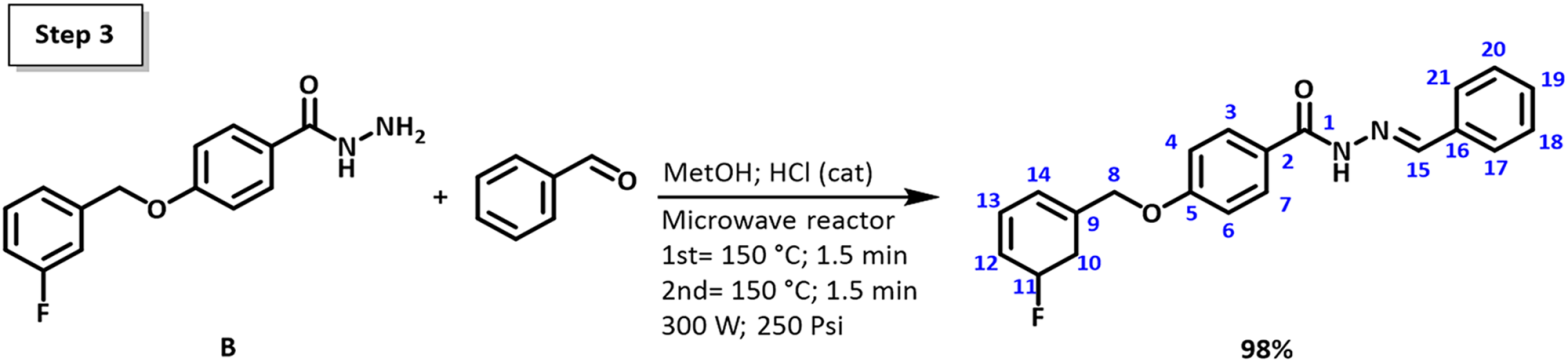
Synthesis of (E)-N'-benzylidene-4-((3-fluorobenzyl)oxy)benzohydrazide (C21H17FN2O2) (FBHZ).

In recent decades, supramolecular chemistry and the understanding of the linear and non-linear optical properties found in crystal molecular assembly have emerged as an important research topic in the rational design of functional solids[1]. In the solid state context, *N*-Acylhydrazones are important systems to study in view of their conformational versatility and potential applications for polymers and for pharmaceutical, photographic and organic non-linear optic (NLO) materials[2]. Chemically, they represent a class of azomethine compounds, which can easily be obtained by the condensation of aldehydes or ketones and acylhydrazines in the presence of an acid catalyst[3,4]. However, the presence of appropriate substituent groups can modify the distribution of molecular electrons leading to various properties, such as antitumor[5,6], antiviral[7], antimicrobia[8] and vasodilatory[9] activity. As part of our research, we have synthesized (E)-4-(3-fluorobenzyloxy)-N'-benzylidenebenzohydrazide, a fluoro-*N*-Acylhydrazone derivative FBHZ (Fig 1).

NLO has led to a wide range of applications in recent years, including in photonics, optoelectronics, spectroscopy and photon blockades [10–19]. With the evolution of scientific knowledge and new technological advances in optical communication, data processing and storage[20–27], there have been reports of controllable switches with excellent performance in NLO [28]. This has raised great interest among researchers in various fields of knowledge, such as physics, chemistry, engineering, and biology [17–19,29–31]. One of the issues concerns organic materials with a good response, including a response for third-order susceptibility and governed by the second hyperpolarizability. Such materials offer rich and varied behaviour in relation to a second-order NLO process, due to their larger frequency range.

Investigations using varied methods and materials have become important for the application of modern optics [32–34]. As is well known, the NLO properties of a crystal usually come from a material constituted by long asymmetric molecules in such a way that, when crystalized, they organize themselves into a crystalline structure and thus maintain their original asymmetry, to some extent. However, even when working with a crystal formed by small molecules, as in the present case, one can use certain techniques to get a crystal with reasonable nonlinear efficiency. In this line, *ab initio* calculations are employed to obtain the structural properties of the molecule. When organic crystals are involved, one should take into account the effects of neighbouring molecules for a better description of their NLO properties; however, the calculations of these properties require sophisticated theoretical models that robustly represent the electronic properties of a crystalline environment [35–38].

Organic molecules with good NLO properties are of great importance for optical applications; hence, theoretical models to calculate these properties have been developed in recent years, with reasonable results when compared to the experimental values. As an example, we cite a recent work in which the authors used a polarization model to estimate the NLO properties, *χ*^(1)^ and *χ*^(2)^ of molecular crystals, with significant results when compared with the experimental data [38]. Other embedding schemes have also been designed for molecular crystals [39–43].

In the present work we describe the synthesis and solid-state characterization of an *N*-acylhydrazone derivative, (E)-4-(3-fluorobenzyloxy)-N'-benzylidenebenzohydrazide (FBHZ). From the Single Crystal X-ray Diffraction (SCXRD) analysis, a detailed inspection of molecular assembly reveals the presence of C−H⋯O, N−H⋯O, C−H⋯π (localized) and C−H⋯F contacts in the FBHZ structure. Here we also present an *ab initio* study using a supermolecule approach to polarization to calculate the dipole moment (*μ*), the linear polarizability (α), the first hyperpolarizability (*β*) and second hyperpolarizability (γ) for the FBHZ, exhibiting NLO properties.

## Experimental and computational procedures

### Synthesis

FBHZ was synthesized by a three-step reaction: **Step 1** synthesis of the compound methyl 4-[(3-fluorobenzyl)oxy]benzoate (**A**); S**tep 2** synthesis of the compound 4-[(3-fluorobenzyl)oxy]benzohydrazide (**B**) (Steps 1 and 2 see S1 Fig). (Fig 1) shows the **Step 3** synthesis of the compound (E)-4-(3-fluorobenzyloxy)-N'-benzylidenebenzohydrazide (FBHZ): **B** (0.260g, 1.0 mmol) was dissolved in methanol (2 mL), followed by addition of 3-fluorbenzaldehyde (1.0 mmol) and a single drop of hydrochloric acid (HCl 37%). The mixture was heated for two cycles of 1.5min at 150°C in a microwave reactor. The reaction was monitored by Thin-layer chromatography (TLC) and, after verifying product formation, the reaction was cooled and the solvent removed by using the vacuum rotary evaporation system. Yield 98%. Molecular formula: C21H17FN2O2 (348.38 g.mol^-1^).

### Single crystal X-ray analysis

The single-crystal X-ray diffraction data for FBHZ were collected at 296(2) K using an APEX2[44] diffractometer with MoKα radiation (λ = 0.71073 Å). The cell refinement and data reduction were carried out also using the software SAINT[45]. The structure was solved by direct methods using SHELXS-2013[46] and anisotropically refined with full-matrix least-squares on F^2^ using SHELXL.[47] The hydrogen atoms on the carbon atoms were positioned geometrically and refined through the riding model [C-H(aromatic) = 0.93 Å with Uiso(H) = 1.2Ueq(C); C-H_2_ = 0.98 Å, with Uiso(H) = 1.2 Ueq(C); N-H = 0.86 (0.92) Å, with Uiso(H) = 1.2 Ueq(N)]. Molecular representation, tables and pictures were generated by Olex2,[48] ORTEP-3[49] and Mercury (version 3.8)[50,51] programs. The UNI intermolecular potential force field (FF)[52,53] was carried out by using via Mercury (version 3.8),[50,51] providing information about energies of strongest lattice contributions coming from inter-contacts. Possible hydrogen bonds were evaluated by using the PARST routine[54] and through the supramolecular features in crystal packing[55–59]. Crystallographic information files were deposited in the Cambridge Structural Data Base[60] under the code CCDC 1497913. Copies of the data can be obtained, free of charge, via www.ccdc.cam.ac.uk.

### Hirshfeld surface analysis

The Hirshfeld surface (HS) and its associated 2D fingerprint plot were performed using Crystal Explorer 3.1[61,62] by constraining these calculations in Density Functional Theory (DFT) at level Becke88/LYP/6-311G(d,p) to experimental X-ray diffraction data (from single crystal FBHZ) via Tonto.[63,64] The surface was generated on the basis of the normalized contact distances, which are defined in terms of *d*_*i*_ (the distance to the nearest nucleus within the surface) and *d*_*e*_ (the distance from the point to the nearest nucleus external to the surface) relative to van der Waals radii[65,66] of the atoms. The high resolution default of *d*_*norm*_ surface (volume: 424.80 Å^3^ and area: 399.79 Å^2^) was mapped over the colour scale, ranging from −0.369 (red) to 1.201 Å (blue), with the fingerprint plots using the expanded 0.6–2.8 Å view of *d*_*e*_ *vs*. *d*_*i*_.

### Thermal analysis

Hot-stage microscopy was performed on a Leica DM2500P microscope connected to the Linkam T95-PE hot-stage equipment. Data were visualized with the Linksys 32 software for hot-stage control. The crystals of FBHZ were placed on a 13mm glass coverslip and put on a 22mm-diameter pure silver heating block inside the stage. The sample was heated at a ramp rate of 10°C.min^-1^ up to a final temperature of 195°C, but discontinued when all material melted. Thermogravimetric analyses (TGA) were evaluated by using a NETZSCH TG 209F1, at a heating rate of 10°C.min^-1^, under nitrogen atmosphere and at a temperature range of 25 to 400°C. The Differential Scanning Calorimetric (DSC) measurements were carried out in a NETZSCH DSC 204 at a temperature range of 25 to 400°C and a heating rate of 10°C.min^-1^ under nitrogen atmosphere, using 2.040 mg of sample mass.

### Theoretical calculations

**Fig 2:**
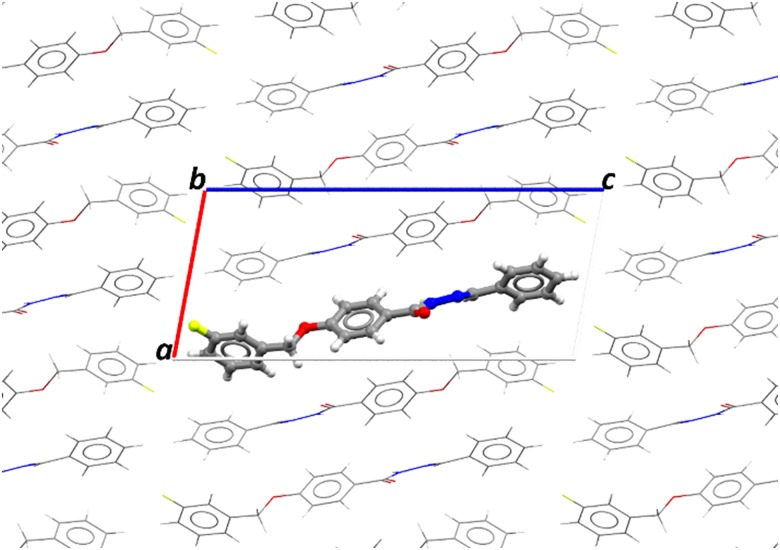
Projection along axis b of the crystal showing the asymmetric unit of embedded FBHZ in the polarization field, the atoms of the molecules of the involved units being treated as point charges.

**Fig 3:**
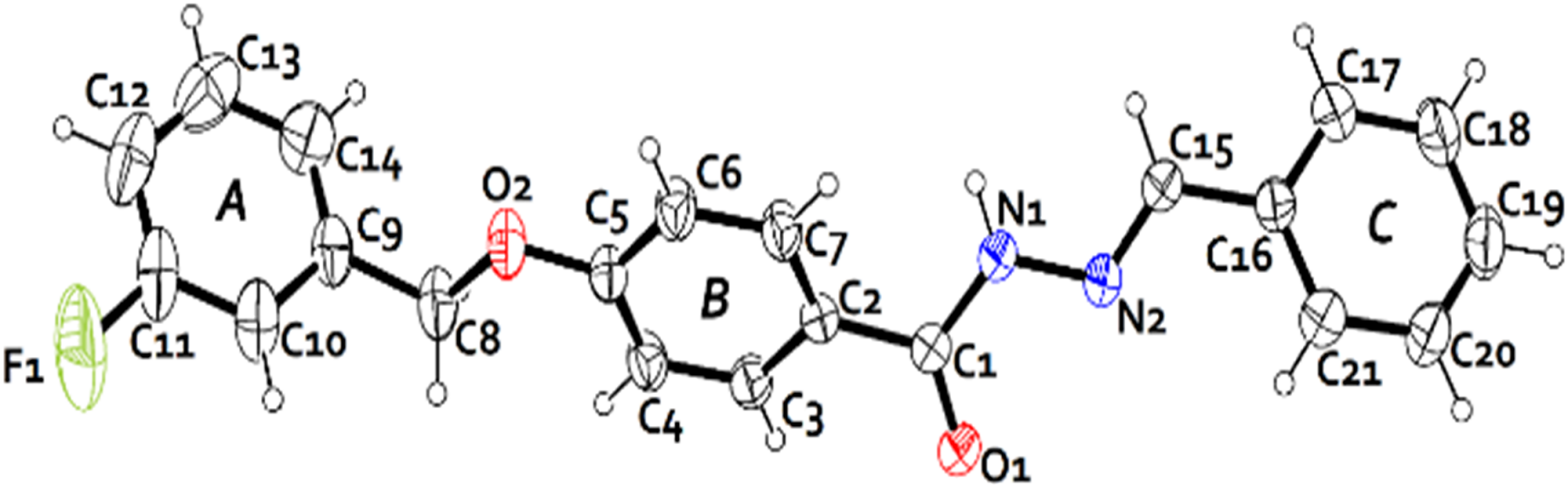
The molecular structure of the FBHZ showing the atom-labelling scheme. Thermal ellipsoids drawn at the 30% probability level. The hydrogen atoms are shown as arbitrary spheres.

In this work, we took advantage of a supermolecule approach that allows us to analyse the polarization effects of the environment on the electrical properties of FBHZ. In this method, the atoms of surrounding molecules are seen as point charges, as shown in (Fig 2), in which each FBHZ molecule (ball and stick) is surrounded by other equal units (wireframe). For the FBHZ a set of 9×9×9 unit cell was used, with 2 asymmetric units in each unit cell, totalizing 1458 unit cells with 62694 atoms, 43 of them forming the FBHZ molecule (Fig 3) to be engaged by the rest. The atoms that form the molecules that surround the FBHZ isolated are treated as point charges, since the interactions between molecules have a dominant electrostatic nature, taking into account long-range electrostatic effects [67,68]. Using the iterative process in which the electrical polarization effects are considered, we have as a major goal the determination of the linear and nonlinear electrical properties of the isolated and embedded FBHZ forming the crystal.

To this end, we started (step zero) with the isolated molecule, via a fit of the electrostatic potential (ChelpG) obtained from the 6–311+G(d) basis set at the MP2 level, described by its charge distribution in a vacuum. Then (i) we calculated the partial atomic charges (ChelpG) for a single isolated molecule of the asymmetric unit; (ii) in the position of each corresponding atom in the generated unit cells, we replaced the atom with its partial atomic charge obtained in item (i); (iii) we calculated the static electric properties (dipole moment and second hyperpolarizability), as well as the new partial atomic charges of the asymmetric unit; (iv) we returned to item (ii) and repeated the procedure until convergence in the electric molecular properties is achieved, as implemented in previous works using this method [36–38].

These calculations showed that a set of 5x5x5 unit cells guarantees fast convergence of the dipole moment of the crystal, so we used a larger arrangement (9x9x9) to ensure a stable value of the dipole moment. The Gaussian-09 program was used in all calculations.

The numerical evaluation employs the following definitions, e.g., the total dipole moment *μ*,

$$\mu =\sqrt{\mu_{x}^{2}+\mu_{y}^{2}+\mu_{z}^{2}}\;,$$ (1)

the linear average polarizability 〈*α*〉,

$$\langle \alpha \rangle =\frac{1}{3}\sum_{i=1}^{3}\alpha_{ii},$$ (2)

the total intrinsic first hyperpolarizability *β*_*Tot*_ defined as

$$\beta_{Tot}=\sqrt{\left(\beta_{x}^{2}+\beta_{y}^{2}+\beta_{z}^{2}\right)},$$ (3)

where

$$\beta_{i}=\frac{1}{3}\sum_{j}\left(\beta_{ijj}+\beta_{jij}+\beta_{jji}\right).$$ (4)

The experimentally relevant quantity is

$$\beta_{\left|\right|}=\frac{1}{5}\sum_{i=1}^{3}\left(\beta_{zii}+\beta_{izi}+\beta_{iiz}\right),$$ (5)

where z is oriented in the direction of the dipole moment of molecule. Sometimes the equivalent quantity $\beta_{vec}=\frac{5}{3}\beta_{\left|\right|}$ is used. The second average hyperpolarizability [69], is given by

$$\langle \gamma \rangle =\frac{1}{15}\;\sum_{ij=x,y,z}\begin{array}{c} \left(\gamma_{iijj}+\gamma_{ijij}+\gamma_{ijji}\right) \end{array}.$$ (6)

The macroscopic linear parameter *χ*^(1)^, second-order nonlinear *χ*^(2)^ and third-order nonlinear susceptibilities *χ*^(3)^ of the crystals can be estimated in a first approximation via the following relations,

$$\chi_{ij}^{\left(1\right)}=\frac{\alpha_{ij}}{V},$$ (7)

$$\chi_{ijk}^{\left(2\right)}=\frac{\beta_{ijk}}{2V},$$ (8)

and,

$$\chi^{\left(3\right)}=\frac{N}{V}\langle \gamma \rangle ,$$ (9)

where N stands for the number of atoms in the unit cell, and V represents its volume Table 1; the dipole moment Table 2; the components *α*_*ij*_ (i, j stand for x, y, z) of the linear polarizability are given in Table 3; *ε*_0_ is the vacuum permittivity and it was built into the Eqs (10), (11) and (12), the components *β*_*ijk*_ of the first hyperpolarizability are given in Table 4, with i, j, k, standing for x, y, z. The values of 〈γ〉 for the isolated and embedded molecules are given in Table 5. The conversion of unit between *χ*^(*n*)^ and α, β and γ are made based on the following relationships,

$$\chi^{\left(1\right)}\left[SI\right]=4\pi \chi^{\left(1\right)}\left[esu\right],\;\;\;and\;\;\alpha \left[\;SI\right]=4\pi \times 10^{-6}\;\alpha \left[esu\right],$$ (10)

$$\chi^{\left(2\right)}\left[\frac{m}{V},SI\right]=\frac{4\pi \times 10^{-4}}{3}\chi^{\left(2\right)}\left[esu\right],\;\;\;and\;\;\beta \left[\frac{m^{4}}{V},\;SI\right]=\frac{4\pi \times 10^{-10}}{3}\beta \left[esu\right],$$ (11)

and,

$$\chi^{\left(3\right)}\left[\frac{m^{2}}{V^{2}},SI\right]=\frac{4\pi \times 10^{-8}}{9}\chi^{\left(3\right)}\left[esu\right],\;\;\;and\;\;\gamma \left[\frac{m^{5}}{V^{2}},\;SI\right]=\frac{4\pi \times 10^{-14}}{9}\gamma \left[esu\right].$$ (12)

**Table 1:** Crystallographic information on the FBHZ.

Identification code	CCDC 1497913	
Empirical formula	C_21_H_17_FN_2_O_2_	
Formula weight	348.36	
Temperature (K)	296(2)	
Crystal system	Monoclinic	
Space group	P2_1_	
Unit cell dimensions	a = 8.5766(5) Å	α = 90°
	b = 5.1893(3) Å	β = 100.451(2)°
	c = 19.7867(10) Å	γ = 90°
Volume (Å^3^)	866.03(8)	
Z	2	
ρ_calc_ (g/cm^3^)	1.336	
μ/mm^-1^	0.774	
F(000)	364.0	
Crystal size (mm^3^)	0.1 × 0.1 × 0.1	
Radiation	CuKα (λ = 1.54184)	
2Θ range for data collection (°)	9.09 to 136.902	
Index ranges	-10 ≤ h ≤ 10, -6 ≤ k ≤ 5, -23 ≤ l ≤ 23	
Reflections collected	10407	
Independent reflections	3088 [R_int_ = 0.0436, R_sigma_ = 0.036]	
Data/restraints/parameters	3088/1/236	
Goodness-of-fit on F^2^	1.046	
Final R indexes [I> = 2σ (I)]	R_1_ = 0.0388, wR_2_ = 0.1071	
Final R indexes [all data]	R_1_ = 0.0502, wR_2_ = 0.1117	
Largest diff. peak/hole (e Å^-3^)	0.18/-0.21	
Flack parameter	0.13(9)	

**Table 2:** MP2/6-311+G(d) results for the components of the dipole moment (D) as function of the iterative process.

FBHZ	*μ*_*x*_	*μ*_*y*_	*μ*_*z*_	*μ*
Isolated	1.67	4.73	-0.15	5.02
Embedded	1.95	3.89	-0.43	4.37
Δ%	16.77	17.76	186.67	12.95

**Table 3:** MP2/6-311+G(d) results for the linear polarizability (10^−24^) esu).

FBHZ	*α*_*xx*_	*α*_*xy*_	*α*_*yy*_	*α*_*xz*_	*α*_*yz*_	*α*_*zz*_	$\bar{\alpha }$
**Isolated**	28.76	1.17	30.59	−11.97	1.07	55.54	38.30
**Embedded**	28.83	1.01	30.66	−12.15	1.27	55.93	38.47
**Δ%**	0.24	13.68	0.23	1.50	18.69	0.70	0.44

**Table 4:** MP2/6-311+G(d) results for the first hyperpolarizability (10^−30^ esu).

	Isolated	Embedded	Δ%
***β***_***xxx***_	0.21	-0.15	171.43
***β***_***xxy***_	-0.25	-0.09	64.00
***β***_***xyy***_	0.20	-0.02	110.00
***Β***_***yyy***_	0.56	2.02	260.71
***β***_***xxz***_	1.29	1.49	15.50
***β***_***xyz***_	0.99	1.13	14.14
***Β***_***yyz***_	-0.47	-0.31	34.04
***β***_***xzz***_	-2.64	-3.16	19.70
***Β***_***yzz***_	-1.60	-1.54	3.75
***Β***_***zzz***_	9.85	11.00	11.68
***Β***_***Tot***_	10.98	12.64	15.12

**Table 5:** Linear optical susceptibility tensor components χ^(1)^ of FBHZ.

FBHZ	$\chi_{xx}^{\left(1\right)}$	$\chi_{xy}^{\left(1\right)}$	$\chi_{yy}^{\left(1\right)}$	$\chi_{xz}^{\left(1\right)}$	$\chi_{yz}^{\left(1\right)}$	$\chi_{zz}^{\left(1\right)}$
	0.42	0.01	0.44	−0.18	0.02	0.81

## Results and discussion

### Synthesis and spectroscopic characterization

The FBHZ was synthesized by the condensation reaction [3], following the three-step reaction herein described. Solid obtained characteristics: yield 98%; molecular formula C_21_H_17_FN_2_O_2_ (348.38 g.mol^-1^); light beige colour; melting point 193–195°C. Single crystals (plates) of FBHZ suitable for X-ray diffraction studies were obtained by slow evaporation from acetone solution at room temperature (~25°C). ^1^H NMR (500 MHz, DMSO-d6) *δ*: 5.23 (s, 2H – 8) 7.15 (m, 3H–12, 6 and 4) 7.32 (d, 2H–14 and 10) 7.45 (m, 4H–13, 18, 19 and 20) 7.72 (d, 2H–17 and 21) 7.91 (d, 2H–7 and 3) 8.45 (s, 1H–15) 11.74 (s, 1H –NH) (S4 Fig). ^13^C NMR (125 MHz, DMSO-6d) *δ*: 99.67 (C-8) 105.77 (C-12, C-10, C-6 and C-4) 107.35 (C-14) 107.99 (C-2) 109.26 (C-17 and C-21) 111.93 (C-18 and C-21) 115.20 (C-3 and C-7) 115.72 (C-13) 121.92 (C-19) 122.13 (C-16) 122.41 (C-9) 134.80 (C-15) 135.07 (C-11) 143.58 (C-1) 151.69 (C-5) (S5 Fig). IR (4000–400 cm^-1^): (C-O) 1183 and 1251; (C-F) 1363; (C = N) 1532; (C = O) 1642; (N-H); 3254 (S2 Fig). MS (m/z): [M+] 348; Fragments: 239; 229; 119; 109 (S3 Fig). Maximum absorbance of 0.805Abs at λ = 302nm on UV scan with 3.2.10^-2^mg.mL^-1^ of sample in ethanol 99.9% (S6 Fig).

### Solid state characterization

**Fig 4:**
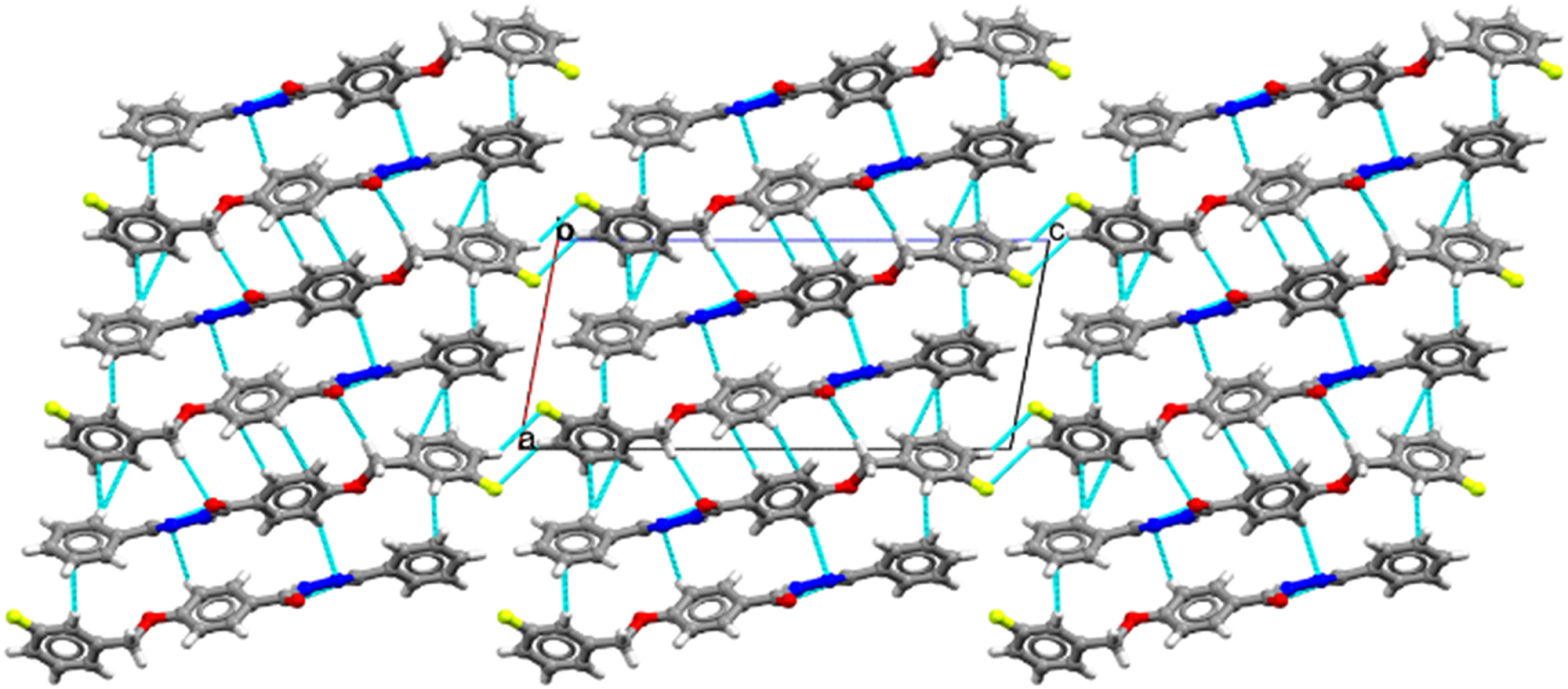
Crystal packing diagram of FBHZ. Cyan dashed lines indicate all possible contacts.

The characterization of FBHZ was performed using SCXRD and thermal analysis. The compound crystallizes in the non-centrosymmetric space group P2_1_ with a single molecule in the asymmetric unit (ASU) (Z' = 1) (Fig 3). The crystal packing of FBHZ is shown in (Fig 4) and the crystal data are given in Table 1.

Additional crystallographic information is present in S6 Fig and S1–S5 Tables. Conformational features were noted through angles (∠, °) between planes and fragment dihedral angles (τ, °) for the FBHZ, which assumes a non-planar geometry from all three aromatic ring planes: ∠A−B = 75.57° [τ(*C5*−*O2*−*C8*−*C9*) = -175.97(4°]; ∠B−C = 69.05° [τ(*C16*−*C15−N2*−*N1*) = -179.39(8°, τ(*O1*−*C1*−*N1*−*N2*) = -4.76(5°, τ(*C1−N1*−*N2*−*C15*) = 168.67(8° and τ(*C2*−*C1*−*N1*−*N2*) = 174.59(8°] and ∠A−C = 12.54°.

**Fig 5:**
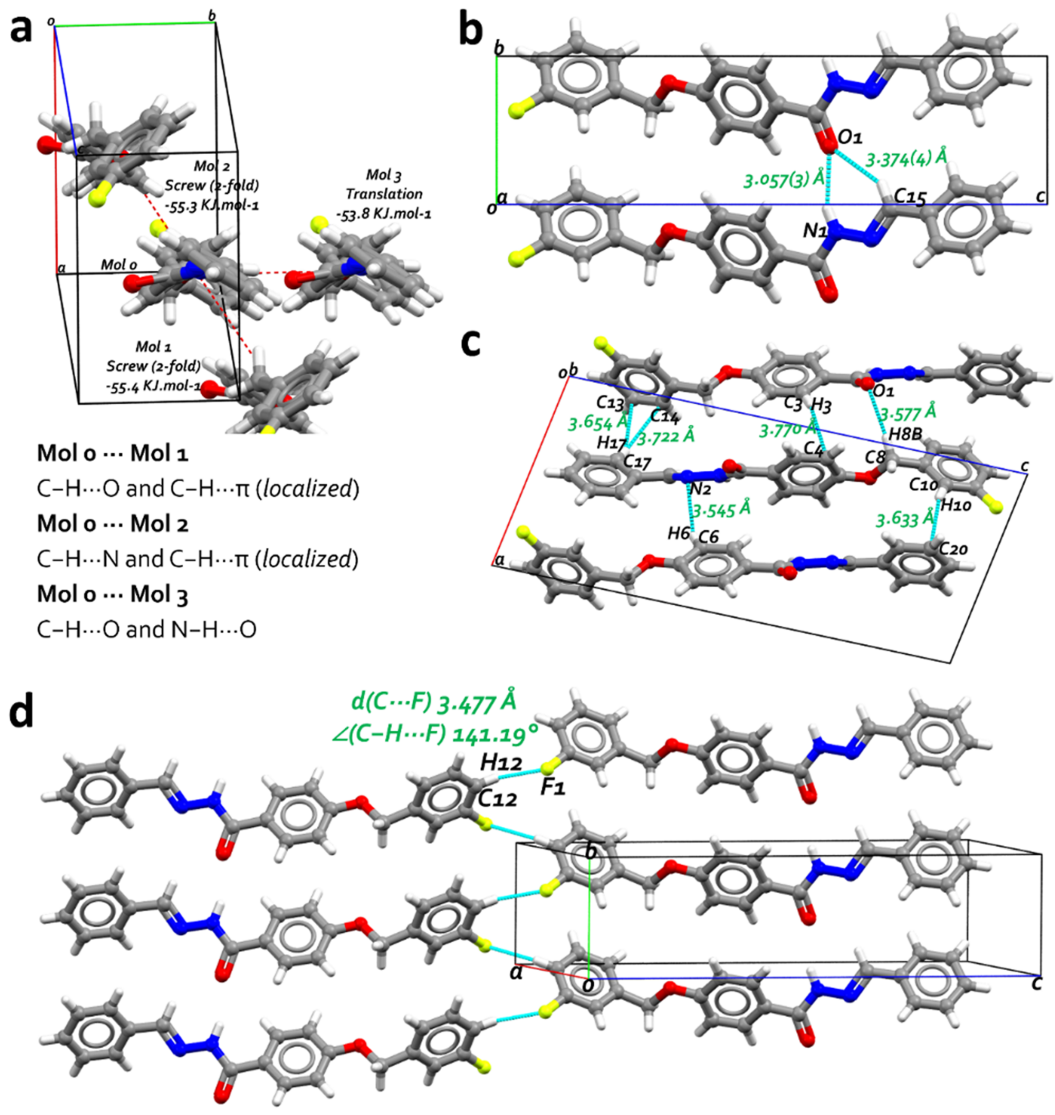
a) UNI intermolecular potential calculation results, showing the three largest lattice contributions of FBHZ in red dashed lines from molecule 0 to each independent molecule [Mol 1: -55.4 kJ.mol-1; Mol 2: -55.3 kJ.mol-1; and Mol 3: -53.8 kJ.mol-1]. b) Bifurcated C−H⋯O/N−H⋯O hydrogen bonds with donor-acceptor distances [d(D⋯A) Å]. c) weak hydrogen bonds C−H⋯O/N−H⋯O and C−H⋯π (localized) contacts with donor-acceptor distances [d(D⋯A) Å]. d) The non-conventional C−H⋯F hydrogen bonds with donor-acceptor distances [d(D⋯A) Å] and C−H⋯F angles [∠(C−H⋯F) °].

Further support for the interactions in the supramolecular arrangement comes from values calculated by the UNI intermolecular potential force field (FF) approach,[52,53] as implemented in Mercury (version 3.8).[50,51] The graphical view of the results (Fig 5a) underlines the fact that the three strongest lattice contributions are between ASU (mol 0) and each of the molecules in the crystal packing (mol 1, mol 2 and mol 3). Inter-fragment energies[70,71] are: -55.4 kJ.mol^-1^ [mol 0 to mol 1 –screw (2-fold)], -55.3. kJ.mol^-1^ [mol 0 to mol 2 –screw (2-fold)] and -53.8 kJ.mol^-1^ [mol 0 to mol 3 –translation]. These calculations also provide the total packing energy (-199.6 kJ.mol^-1^).

In the crystal structure, a $C_{2}^{1}\left(6\right)$ motif is formed by the C15−H15⋯O1 [d_*C15⋯O1*_ = 3.374(4) Å] and N1−H1⋯O1 [d_*N1⋯O1*_ = 3.057(3) Å] hydrogen bonds. As a consequence, molecules are arranged into a 1D-chain along the [10] direction (Fig 5b). Adjacent chains are 2D assembled (Fig 5c) by the formation of the C17−H17⋯π (π localized on C13 and C14) [d_*C17⋯C13*_ = 3.654(5) Å; d_*C17⋯C14*_ = 3.722(5) Å], C3−H3⋯π (π localized on C4) [d_*C3⋯C4*_ = 3.770(5) Å], C8−H8B⋯O1 [d_*C8⋯O1*_ = 3.576(4) Å], C6−H6⋯N2 [d_*C6⋯N2*_ = 3.545(4) Å], C10−H10⋯π (π localized on C20) [d_*C10⋯C20*_ = 3.633(5) Å] weak hydrogen bonds[56,58,72–76]. The 3D assembly is completed considering a weak hydrogen bond C−H⋯F[77–80] between C12−H12⋯F1 [d_*C12⋯F1*_ = 3.477(6) Å], which holds together the molecules along the [1] direction (Fig 5d), and the existence of these combined interactions supports the supramolecular assembly.

**Fig 6:**
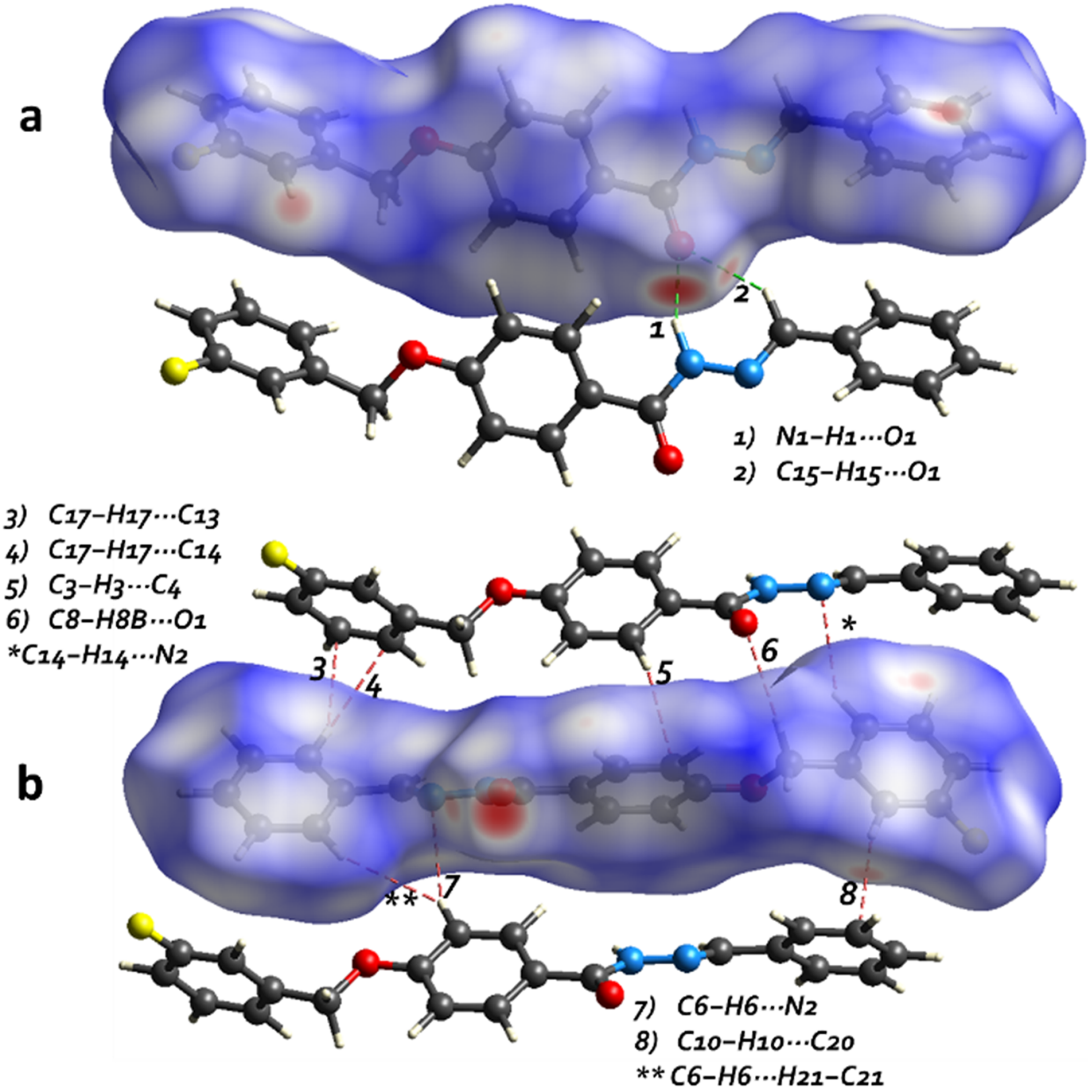
The HS mapped with dnorm property, highlighting regions of contact such as H⋯O (C−H⋯O/N−H⋯O hydrogen bonds), H⋯N (C−H⋯N weak hydrogen bonds) and H⋯C [C−H⋯π (localized on C)] contacts.

Hirshfeld Surface (HS) analysis was performed to identify important intermolecular interactions and their quantitative contributions to the stability of the supramolecular assembly of FBHZ. Furthermore, the analysis confirms the existence of weak interactions disclosed by computational methods. The (Fig 6a and 6b) presents the *d*_*norm*_ maps of FBHZ with interaction areas (red) located on the O-atom of carbonyl (C = O), H-atoms of aromatic (C-H) π-localized and N-atoms of the (HN-N = C) azo group. These red spots observed corroborate the regions of contact in the supramolecular arrangement.

**Fig 7:**
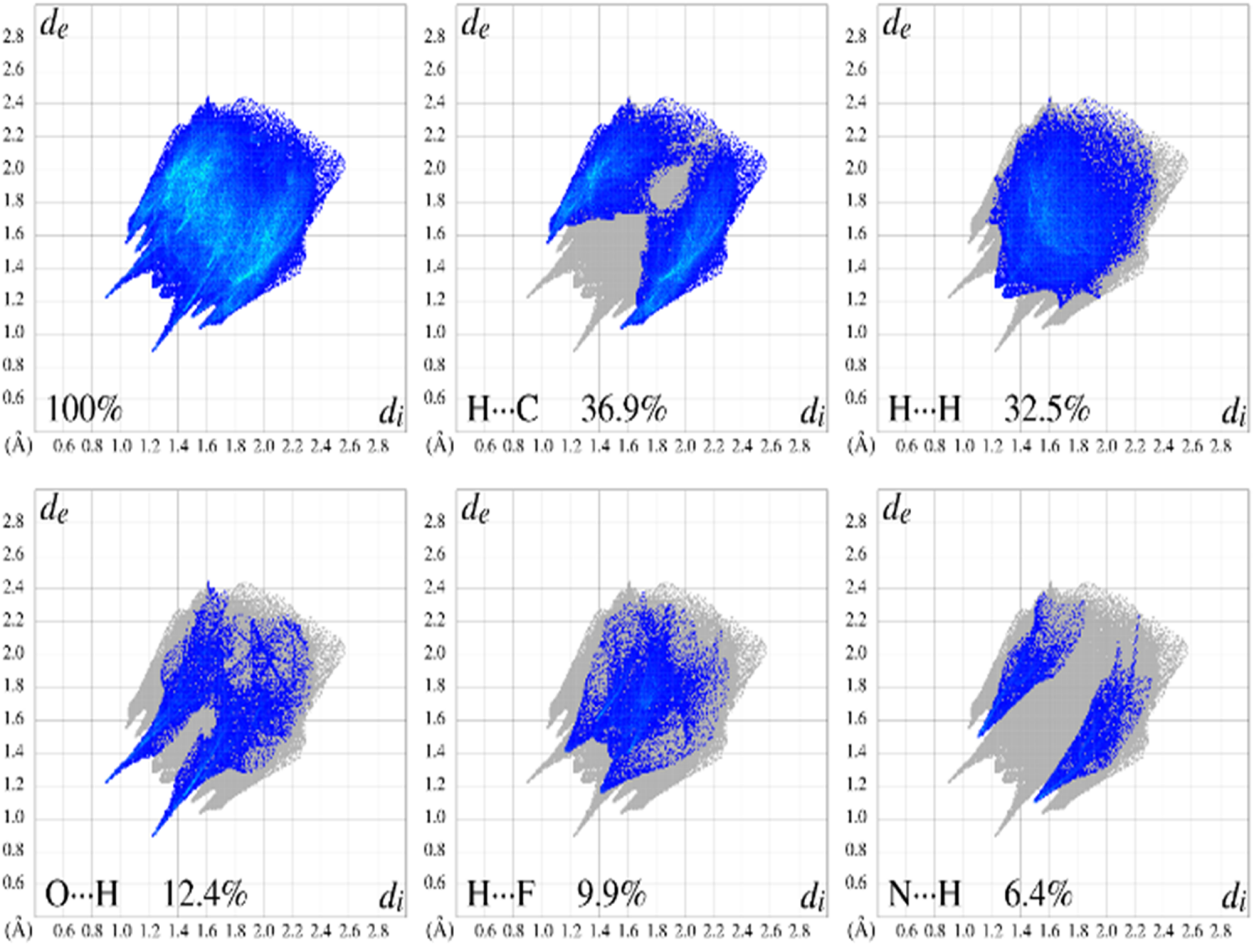
2D fingerprint plots showing the percentage decomposition from each indicatory type of contact related to C−H⋯π [H⋯C 36.9%], C−H⋯O [O⋯H 12.4%], C−H⋯F [H⋯F 9.9%], N−H⋯O [N⋯H 6.4%] and H⋯H 32.5% in HS for FBHZ.

The associated *d*_*e*_ *vs*. *d*_*i*_ fingerprint plots summarize the contributions of intermolecular contacts to the total HS area of FBHZ (Fig 7). The two peaks at 1.4–1.2 Å *d*_*i*_+*d*_*e*_ are associated with O⋯H contacts (12.4%) and attributed to C−H⋯O/N−H⋯O interactions of the 1D and 2D assembly of FBHZ. There is evidence of C−H⋯π (localized) contacts in the structure associated with H⋯C contacts (36.9%), the largest value from this HS area, which occur as a characteristic peak on the side borders of the plot at 1.5–2.6 Å *d*_*i*_+*d*_*e*_. These contacts happen around the hydrophobic region, in this case in aromatic groups.

The HS analysis of FBHZ molecules shows the second largest contribution from H⋯H type contacts, contributing to 32.5% of the total area. This is in accordance with the strong contribution of aromatic groups to the formation of hydrophobic regions where various *van der* Waals contacts are formed. Noticeable contributions from H⋯F and N⋯H contacts are also observed, showing a percentage of 9.9% and 6.4% of HS area, respectively. H⋯F is associated with C−H⋯F and N⋯H to C−H⋯N weak hydrogen bonds, as previously discussed.

**Fig 8:**
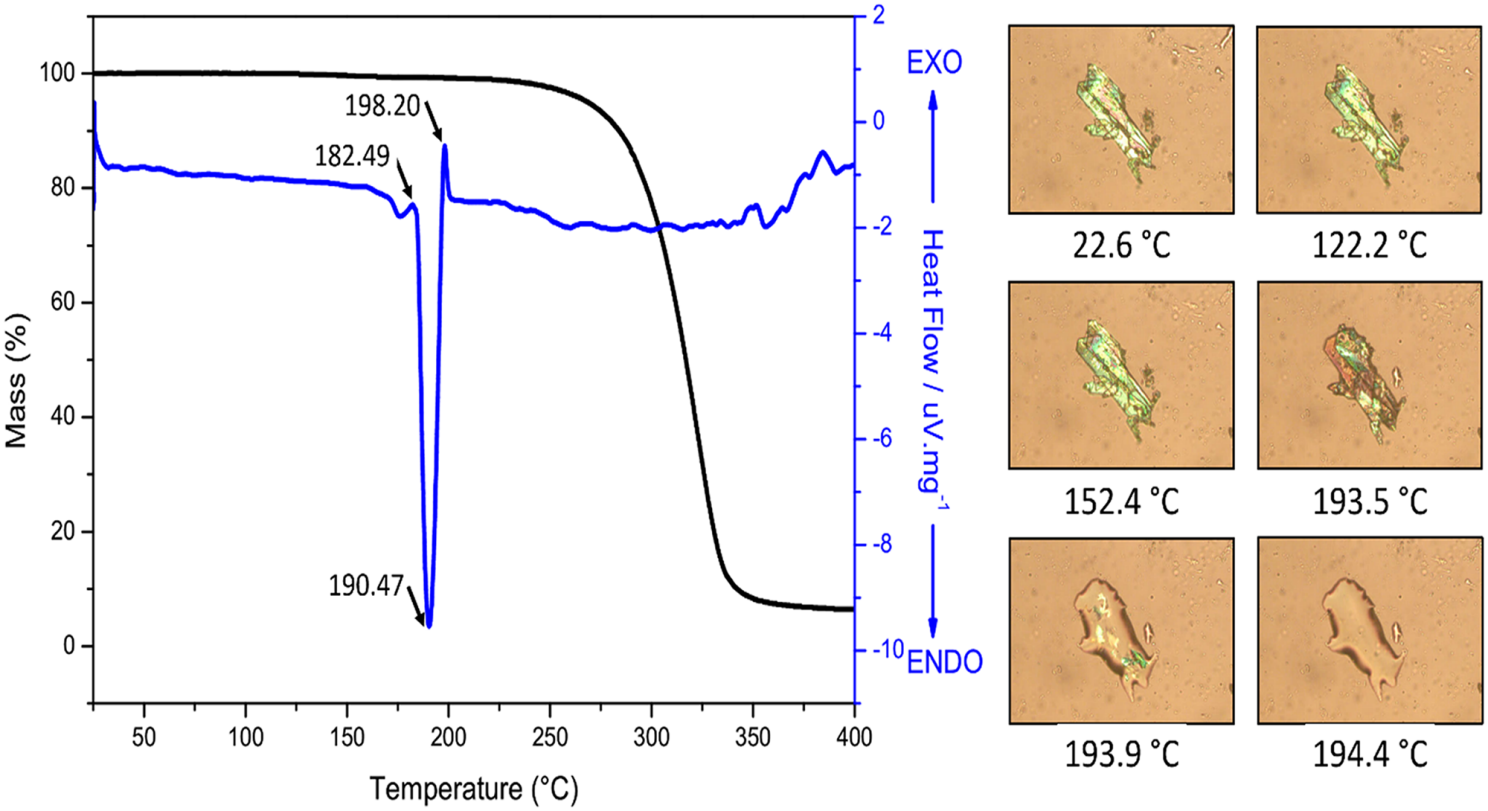
Heating-cooling-reheating DSC and TGA data for the FBHZ. The solid-liquid transition via Hot-stage Microscopy analysis is also shown.

In order to characterize the crystalline structure of FBHZ fully, DSC/TGA and Hot-stage Microscopy analysis were performed, and the results are presented in (Fig 8). The DSC measurement shows only one endothermic event centred at 190.47°C. The TGA curve shows the beginning of weight loss at 224°C. Since no weight loss is detected in the TGA curve until ~220°C, the endothermic event is associated with a solid-liquid phase transition. This transformation is irreversible, since no corresponding event occurs during the cooling-reheating cycles of DSC, and meanwhile the entropy decrease due to the ordering of FBHZ molecules on crystalline state within the system is overcompensated by the thermal randomization of the surroundings. Furthermore, the event at 190.47°C in the DSC curve corresponds to the complete melting of the sample and was confirmed by the Hot-stage Microscopy analysis, which takes close values as in the range 182–198°C. No habit or colour changes are observed for the sample between 22 and 152°C. However, the solid-liquid phase transformation is detected in Hot-stage photography, when the crystals change their colour, becoming an opaque material at ~190°C. The complete melting of the sample is achieved at 194°C.

### Theoretical study of NLO properties

We addressed the evolution of the electrical properties of dipole moment (*μ*), linear polarizability (*α*), first hyperpolarizability (*β*) and second hyperpolarizability (*γ*). We took into account the number of iterations for FBHZ, both isolated and embedded by other molecules in the same structure. The MP2 theory levels were used with the basis set of functions 6–311+G(d) for all electrical properties, with the exception of the second hyperpolarizability, in which the CAM-B3LYP was used, albeit keeping the same basis set functions. Previous studies have shown that the set basis used in this work is recognized as adoptable, [36] since the results for the dipole moment are reasonable in comparison with experimental data [81]. The following tables show the results of these properties of the crystal as a function of the iteration numbers.

**Fig 9:**
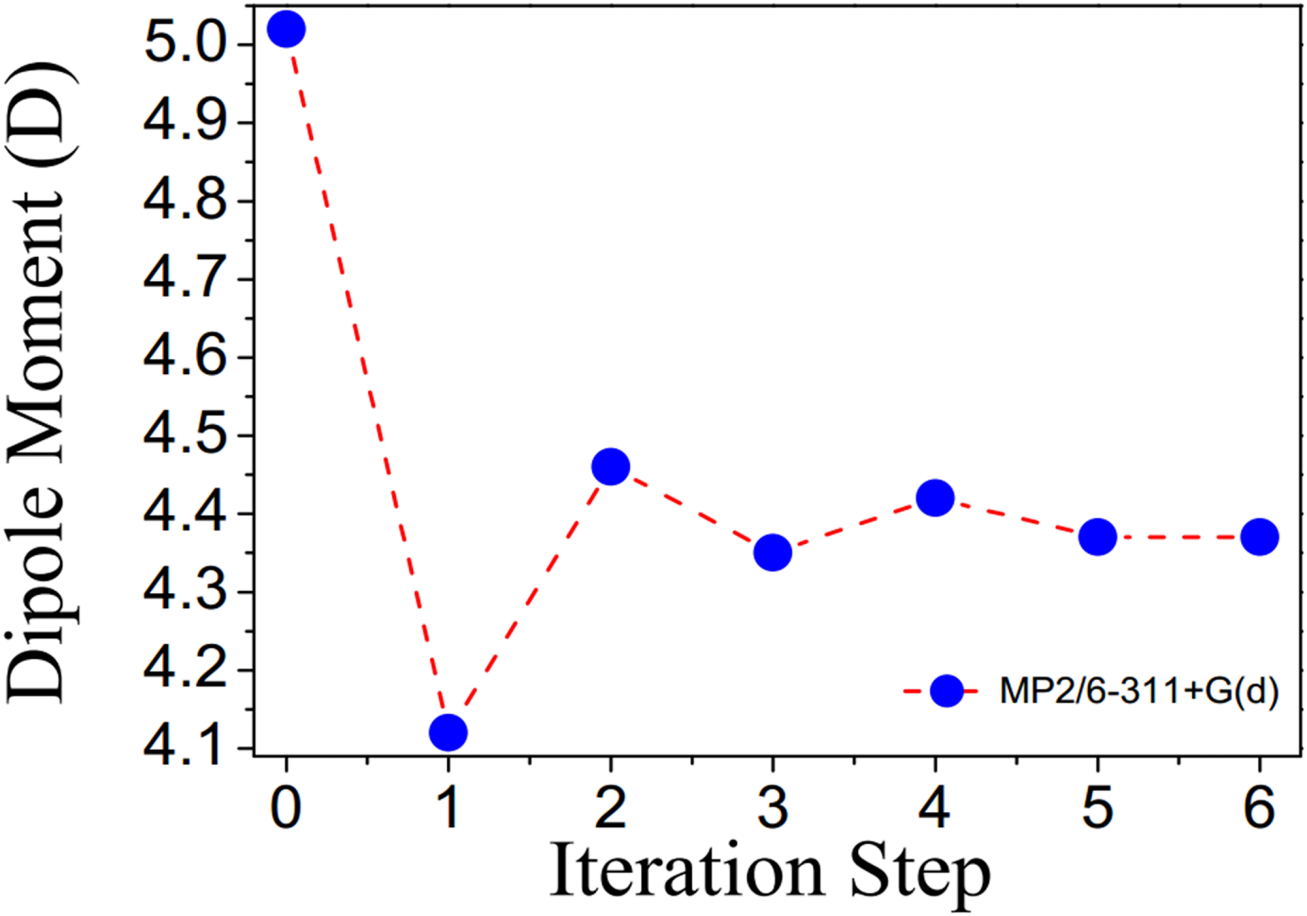
Evolution of values of the dipole moment of the FBHZ with the respective iteration numbers. A 9×9×9 unit cell assembly was considered (step 0 indicates the isolated molecule and the other steps indicate the embedded molecule).

The applicability of the supermolecule approach and the scheme of electrostatic polarization is advantageous due to the rapid convergence of the dipole moment of FBHZ throughout the process, in which six iterations were considered. The convergence of iterative series for this electrical property can be seen in (Fig 9). The value of the total dipole moment (***μ***) for the embedded FBHZ is 4.37 D, showing a decrease of 12.95% when compared with the isolated FBHZ, 5.02 D. Equivalent results for the dipole moment due to crystal field effects have also been reported [37,82,83]. In Table 2, the ***μ***_***y***_ component is shown displaying values closer to the final result, hence providing the greatest contribution to the dipole moment. On the other hand, the ***μ***_***z***_ is the component that provides the least contribution.

The present supermolecule approach, combined with an electrostatic polarization scheme, satisfactorily reproduces the average value of the linear polarizability when compared with experimental results [38]. The calculations of the linear polarizability, shown in Table 3, were implemented via MP2 using the base function set 6–311+G(d). Analysing the second linear property, we note that the value of the linear polarizability 〈*α*〉 for the isolated FBHZ is 38.30 × 10^−24^ esu, whereas for the embedded molecule one finds 38.47 × 10^−24^ esu, showing an increase of 0.44%. Checking the components, we notice them exhibiting different variations, in which ***α***_***xx***_, ***α***_***yy***_, ***α***_***yz***_, ***α***_***zz***_ show a percentage increase, with the component ***α***_***yz***_ exhibiting the greatest variation, 18.69%. On the other hand, the ***α***_***xy***_ is reduced in percentage by 13.68%.

The same method as that applied in the foregoing paragraph also satisfactorily reproduces the value of the first hyperpolarizability in comparison with experimental results [38]. The calculations of the first hyperpolarizability were implemented with the MP2 method, using the base function set 6–311+G(d). Considering now the first nonlinear property, Table 4 shows the first hyperpolarizability values for FBHZ, and the value for the (*β*_*Tot*_) is 10.98 × 10^−30^ esu isolated and 12.64 × 10^−30^ esu embedded, corresponding to an increase of 15.12%. Higher percentages also occur, as found in the ***β***_***xxx***_ 171.43% and ***β***_***xyy***_ 110% components, in which reduction occurs; on the other hand, one finds the components ***β***_***xxy***_ and ***β***_***yyy***_ with 64% and 260.71%, respectively, reporting a percentage increase. The other components also undergo variations due to influences that come from polarization of the environment; but in this case the magnitude is reduced, as happens for ***β***_***Tot***_. We also observed component ***β***_***zzz***_ giving the greatest contribution to the first total hyperpolarizability, ranging in 14.91% (embedded). The first calculated hyperpolarizability of the crystal FBHZ is better than the experimental result for the (1E,4E)-1,5-di-p-tolylpenta-1,4-dien-3-one; our result is 40% higher and becomes an attractive object for future studies in nonlinear optics [84].

The use of the supermolecule approach generally leads to a better description of the Optical Properties. The calculated values *χ*^(1)^ and *χ*^(2)^ are summarized in Tables 5 and 6.

**Table 6:** Second-order nonlinear optical susceptibility tensor components χ^(2)^ (in pm/V) of FBHZ.

*FBHZ*	$\chi_{xxx}^{\left(2\right)}$	$\chi_{xxy}^{\left(2\right)}$	$\chi_{xyy}^{\left(2\right)}$	$\chi_{yyy}^{\left(2\right)}$	$\chi_{xxz}^{\left(2\right)}$	$\chi_{xyz}^{\left(2\right)}$	$\chi_{yyz}^{\left(2\right)}$	$\chi_{xzz}^{\left(2\right)}$	$\chi_{yzz}^{\left(2\right)}$	$\chi_{zzz}^{\left(2\right)}$
	−0.04	−0.02	−0.01	0.49	0.36	0.27	−0.08	−0.76	−0.37	2.66

Table 7 displays the second hyperpolarizability 〈γ〉 in the static case; it presents the value 65.63 × 10^−36^ esu for the isolated FBHZ, while the embedded FBHZ shows a slight increase of 5.12% to the value of 68.99 × 10^−36^ esu. A. N. Castro et al. [37] explained how to increase this percentage, due to the influence of an electrostatic field. Observing all components, we notice they undergo a slight increase in their value, with the exception of the *γ*_*xxxx*_ component, which undergoes a reduction of 3.87%. For the embedded FBHZ, there are N = 86 atoms inside the unit cell and the cell volume V = 866.03 Å^3^; we have found *χ*^(3)^ ≈ 10^−19^ *m*^2^/*V*^2^ (*SI*). This result has the same order of magnitude of the value found for bulk gold[85,86], indicating the reasonability of our result. This demonstrates that FBHZ constitutes a promising object for applications in quantum dots[87].

**Table 7:** CAM-B3LYP/6-311+G(d) results for the second hyperpolarizability (10^−36^ esu) in the static case.

	*γ*_*xxxx*_	γ_yyyy_	γ_zzzz_	γ_xxyy_	γ_yyzz_	γ_xxzz_	〈γ〉
**Isolated**	26.11	17.87	181.26	4.91	9.69	36.86	65.63
**Embedded**	25.10	18.92	194.08	4.98	9.81	38.64	68.99
**Δ%**	3.87	5.88	7.07	1.43	1.24	4.83	5.12

Accordingly, the percentage differences are more significant for the first hyperpolarizability compared to the linear polarizability and second hyperpolarizability. This happens because the polarization effects of the crystalline environment have a greater impact on the components of the tensor representing the first hyperpolarizability, since in general the influence of an electrostatic field is great. One example is the signal change that occurs for the ***β***_***xxx***_ and ***β***_***xyy***_ components, as also reported in earlier studies [36,38].

The dynamic effects of the linear polarizability, the first hyperpolarizability, and the second hyperpolarizability are shown in. The second average hyperpolarizability γ(-2ω;ω,ω,0) is a dc-electric field induced second harmonic generation (EFISH), while the value γ(-ω;ω,0,0) corresponds to the Kerr effect. The linear polarizability α(-ω;ω), the first hyperpolarizability β_||_(-2ω;ω,ω), and the second average hyperpolarizability γ(-2ω;ω,ω,0), for the isolated and embedded FBHZ at frequency ω = 0.042 au, are shown in Table 8. Comparing the value of the α(-ω;ω) and γ(-2ω;ω,ω,0) at frequency ω = 0.0428 au, for the isolated FBHZ in the static cases α(0;0) and γ(0;0,0,0), we note an increase of 3.31% and 44.64%, respectively. When we compare the values of the crystal FBHZ at frequency ω = 0.0428 au of α(-ω;ω) and γ(-2ω;ω,ω,0) in the static cases α(0;0) and γ(0;0,0,0), we have an increase of 3% and 47% respectively. The first hyperpolarizability β_||_(-2ω;ω,ω) at frequency ω = 0.042 a.u. of the crystal FBHZ shows an increase of 17.20% when compared with the isolated FBHZ.

**Fig 10:**
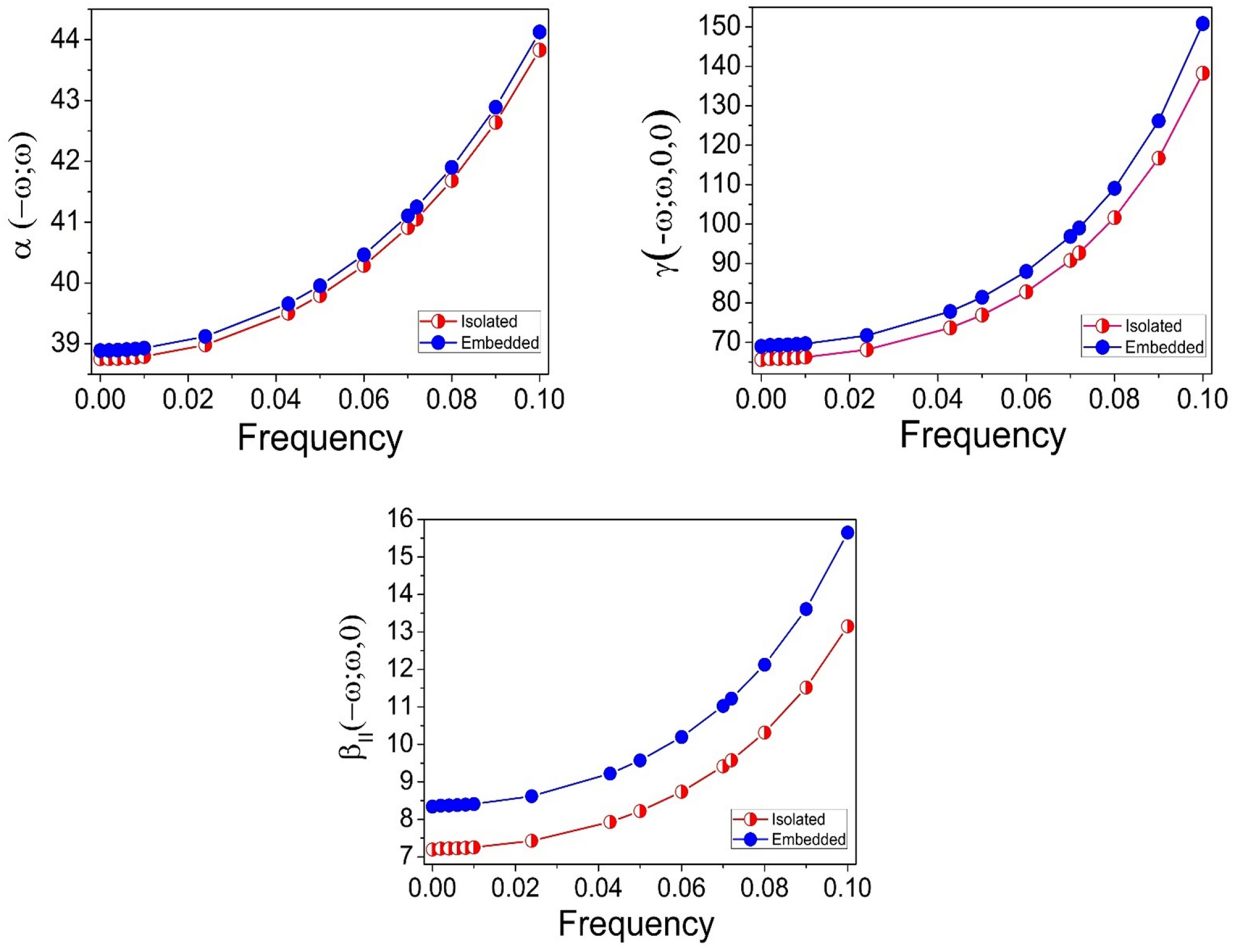
Dynamic evolution of the calculated values for the linear polarizability (10^−24^ esu), first hyperpolarizability (10^−30^ esu), and second hyperpolarizability (10^−36^ esu) of FBHZ with the respective number of frequencies.

The dispersion curves of the linear polarizability and the first and second hyperpolarizabilities for the isolated and embedded FBHZ molecules are shown in (Fig 10). The linear polarizabilities of the embedded and isolated molecules of FBHZ show a similar behaviour as a function of the frequency of an external applied field, the same being true for the first and second hyperpolarizabilities.

**Table 8:** CAM-B3LYP/6-311+G(d) results for the dynamic linear polarizability (10^−24^ esu), first hyperpolarizability (10^−30^ esu), and second hyperpolarizability (10^−36^ esu) of the isolated and embedded FBHZ at frequency ω = 0.0428 a.u.

FBHZ	α(-ω;ω)	β||(-2ω;ω,ω)	γ(-2ω;ω,ω,0)
Isolated	39.50	9.77	94.93
Embedded	39.65	11.45	101.56
Δ%	0.38	17,20	7,00

Table 9 shows the influence of polarization upon the electron density of the FBHZ due to the field of point charges of the atoms of neighbouring molecules, which can also be qualitatively analysed in terms of the partial atomic charges. The results of the CHELPG charges for the isolated and embedded cases show a small charge transfer between C−H⋯F (C12−H12⋯F1). This occurs due to a weak hydrogen bond. Within the dihedral angles of the crystal, the fragment that transfers the greatest charge between the isolated FBHZ and the embedded FBHZ is (*C5*−*O2*−*C8*−*C9*). One reason for the small γ growth and, to a lesser degree, μ and ***β*** in a crystalline environment can be found in the results shown in Table 9. There is a significant charge transfer from ring B (C5-C6-C7-C2-C3-C4) around 41.64%; the conformation (C8-O2) received the greatest charge, around 38.35%.

**Table 9:** MP2/6-311+G d results for the CHELPG atomic charges of isolated and embedded FBHZ.

Number	Atom	Charge (10^−19^*C*)		
		Isolated	Converged	Δ%
1	N2	-0.41	-0.42	2.44
2	O2	-0.75	-0.95	26.67
3	O1	-0.88	-1.01	14.77
4	N1	-0.50	-0.58	16.00
5	H1	0.42	0.49	16.67
6	C5	0.66	0.83	25.76
7	C4	-0.45	-0.48	6.67
8	H4	0.25	0.28	12.00
9	C3	-0.06	-0.05	16.67
10	H3	0.18	0.20	11.11
11	C7	-0.21	-0.21	0.00
12	H7	0.22	0.24	9.09
13	C1	1.02	1.11	8.82
14	C2	-0.23	-0.31	34.78
15	C6	-0.36	-0.44	22.22
16	H6	0.23	0.25	8.70
17	C16	-0.10	-0.17	70.00
18	C15	0.36	0.37	2.78
19	H15	0.12	0.17	41.67
20	C9	0.06	0.13	116.67
21	C19	-0.16	-0.13	18.75
22	H19	0.17	0.18	5.88
23	C17	-0.18	-0.18	0.00
24	H17	0.15	0.14	6.67
25	C8	0.69	0.68	1.45
26	H8A	-0.06	0.02	133.33
27	H8B	-0.10	-0.06	40.00
28	C21	-0.08	-0.07	12.50
29	H21	0.11	0.13	18.18
30	C10	-0.56	-0.55	1.79
31	H10	0.31	0.34	9.68
32	C11	0.73	0.70	4.11
33	C18	-0.11	-0.15	36.36
34	H18	0.16	0.16	0.00
35	C14	-0.31	-0.38	22.58
36	H14	0.23	0.24	4.35
37	C13	-0.09	-0.12	33.33
38	H13	0.18	0.19	5.56
39	F1	-0.47	-0.46	2.13
40	C20	-0.15	-0.17	13.33
41	H20	0.17	0.20	17.65
42	C12	-0.45	-0.44	2.22
43	H12	0.28	0.28	0.00

We also used an implicit solvation scheme, based on PCM integral equation formalism (PCM-IEF). In this case, calculations of the dynamic electrical properties of FBHZ were implemented, but this time including different solvents (Gas-Phase, Acetone, Chloroform, Dichloromethane, Dimethyl sulfoxide, Ethanol, Methanol, and Water). The results can be seen in S6 Table. For the extreme cases, i.e., the gas-phase and dimethyl sulfoxide (DMSO), the linear polarizability and the first and second hyperpolarizability are shown in Table 10. As in the case discussed in Table 8, the values γ(-2ω;ω,ω,0), β_||_(-2ω;ω,ω) and α(-ω,ω) are much more sensitive in the presence of solvents. We can numerically confirm that DMSO increased the value of γ(-2ω;ω,ω,0) by nearly 62.65%, whereas for β||(-2ω;ω,ω) and α(-ω;ω), the increase was 71.93% and 10.82%, respectively.

**Table 10:** CAM-B3LYP/6-311+G(d) results for the dynamic linear polarizability (10^−24^ esu), first hyperpolarizability (10^−30^ esu) and second hyperpolarizability (10^−36^ esu) of gas-phase and DMSO solvent FBHZ for the frequency ω = 0.0428 a.u.

Medium	α(-ω;ω)	β||(-2ω;ω,ω)	γ(-2ω;ω,ω,0)
Gas-Phase	43.62	9.19	129.38
DMSO	48.34	15.80	210.44
Δ%	10.82	71.93	62.65

### Frontier molecular orbitals

The orbital of a molecule can be ordered according to either the energy levels in a diagram, which can be mounted, or the experimental results using computational methods (theoretical calculation). The molecular orbital of highest energy, occupied by at least one electron, is called the *HOMO*, and the molecular orbital of lowest energy not occupied by electrons, the *LUMO* [88,89]. The values of the energy of the HOMO and LUMO for the isolated molecule are −7.74 *eV*, 1.89 *eV* respectively. In accordance with these energies, we can calculate the chemical potential, hardness and electronegativity. The energy difference between HOMO and LUMO (*Band* − *Gap*) was calculated for both isolated and embedded FBHZ: the following values were found: 9.63 *eV* and 9.39 *eV*. The *Band* − *Gap* is an indicator of molecular stability; compounds with a high *Band* − *Gap* are stable [90,91].

The first hyperpolarizability can also be connected to the band gap of energy between the *HOMO* and *LUMO*. So, the higher the *Band* − *Gap* is, the lower the first hyperpolarizability. When we compare the value of the Band-Gap of the isolated molecule with that of the embedded molecule, we note a percentage decrease of 2.5%, which indicates that the embedded molecule has a higher potential for applications in NLO [91].

**Fig 11:**
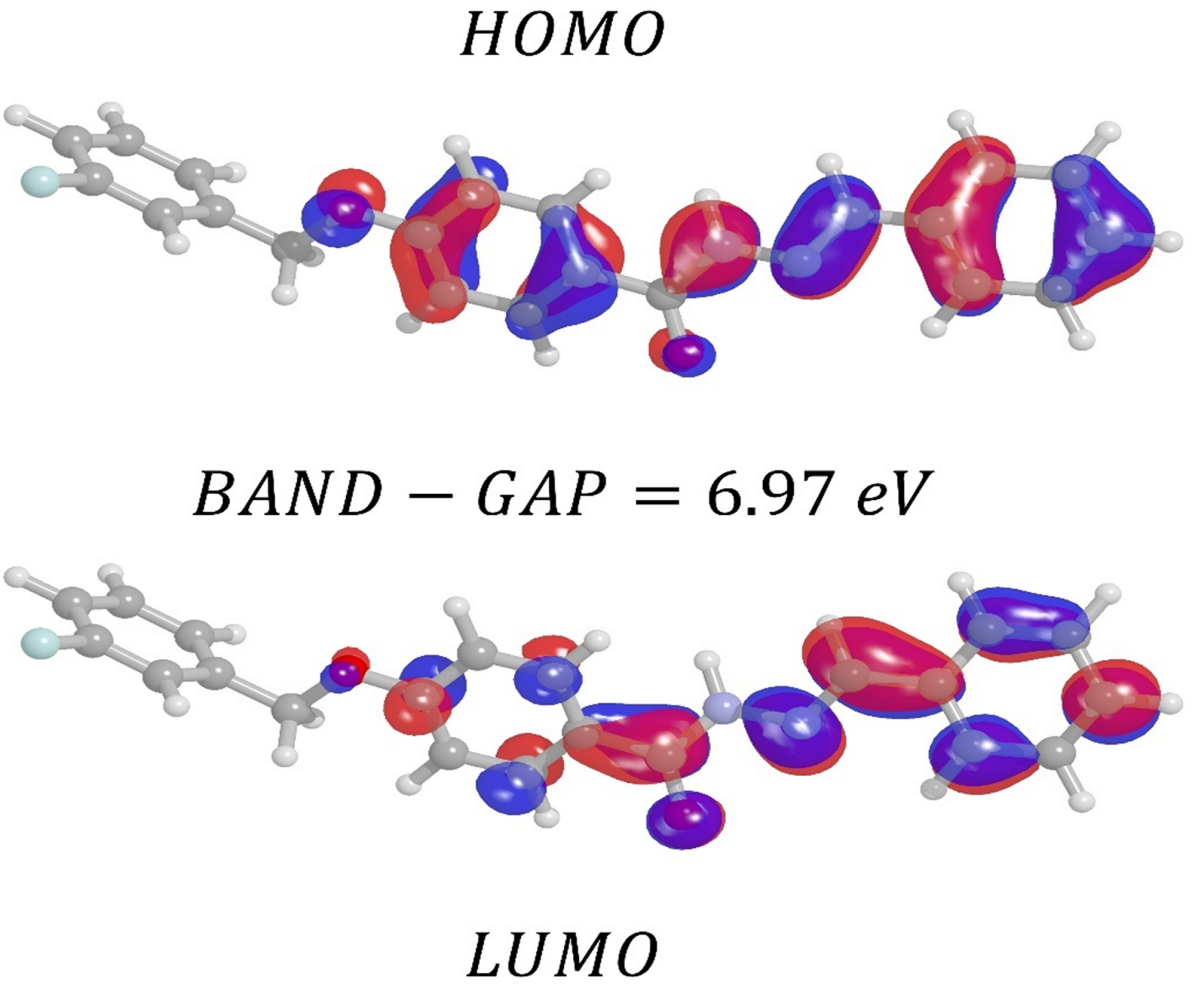
Molecular orbital plots, showing HOMO-LUMO as obtained in the CAM-B3LYP/6-311+G(d) level of theory for DMSO solvent, for the FBHZ molecule.

**Fig 12:**
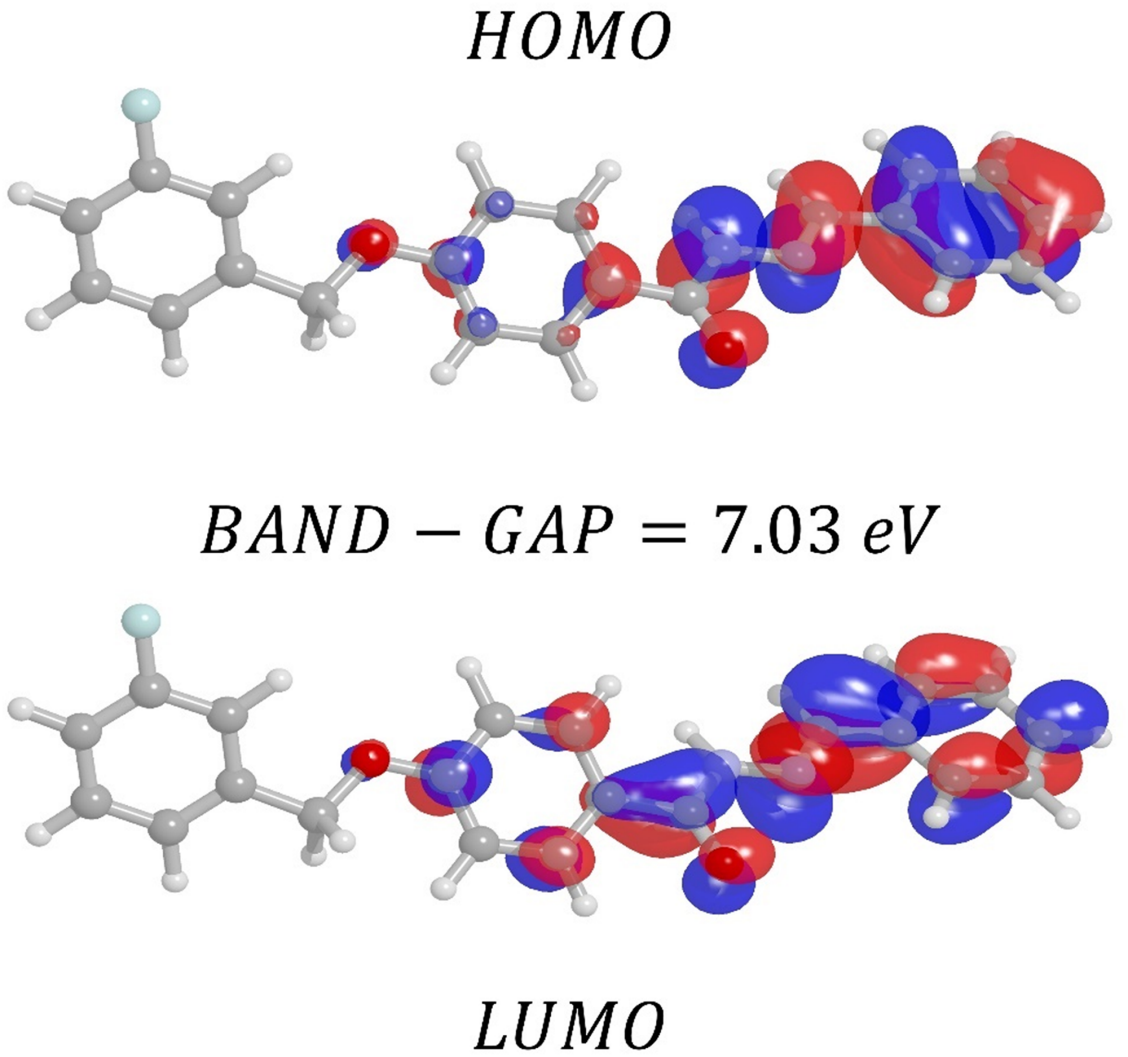
Molecular orbital plots, showing HOMO-LUMO as obtained in the CAM-B3LYP/6-311+G(d) level of theory for gas-phase, for the FBHZ molecule.

Looking at the energy Band-Gap between the *HOMO* and *LUMO* of the molecule FBHZ in a solvent medium, in this case DMSO see (Fig 11) and Gas-Phase see (Fig 12), we note a small Band-Gap of 6.97 eV (DMSO) and 7.03 eV (Gas-Phase), which indicates that crystal FBHZ has promising applications for NLO. The energy Band-Gap between the HOMO and LUMO for different solvents (Acetone, Chloroform, Dichloromethane, Ethanol, Methanol, and Water) can be seen in S8–S13 Figs.

In the results for the gas-phase FBHZ, the oscillator strength of the second state is much weaker compared to the first state, see Table 11. When the calculation is done in DMSO, the weaker state is the second excited state. The transitions reported show that the states change places when going from gas-phase to DMSO and the transition from HOMO to LUMO becomes lower in energy than the transition from HOMO to LUMO+1 in the DMSO system.

**Table 11:** TDDFT PBE1PBE/6-311+G(2d,p) of the FBHZ molecule in the gas phase and DMSO.

	Wavelength (nm)	Energy (eV)	Oscillator Strength
Gas-Phase			
State 1	442.39	2.8026	0.0148
State 2	393.43	3.1513	0.0004
DMSO			
State 1	402.24	3.0824	1.4014
State 2	297.91	4.1618	0.1696

## Conclusions

In this work we have determined the total dipole moment, the linear polarizability and the first and second hyperpolarizabilities of the FBHZ crystal. We employed a supermolecule approach combined with an iterative scheme of electrostatic polarization, where the atoms of neighbouring molecules are represented by point charges. This technique can represent the experimental results very successfully, see Ref. [36,38]. Urea is a prototypical molecule used in the NLO properties of molecular systems. Hence, it was used frequently as a threshold value for the purpose of comparison[92]. Thus we have found that the total dipole moment of the FBHZ embedded molecule is approximately 3.18 times greater than that of Urea while the total first order hyperpolarizability is 40.6 times greater than that of Urea and the second hyperpolarizability is 16.6 times greater than that of Urea. The result of the calculation of first hyperpolarizability of the crystal FBHZ is 40% greater than the experimental result for the (1E,4E)-1,5-di-p-tolylpenta-1,4-dien-3-one[84]. Hence, we can conclude, on the basis of the magnitude of the first order hyperpolarizability of the FBHZ, that it offers potential applications in the development of NLO material. Our results for NLO properties are of the same order of magnitude (*χ*^(3)^ ≈ 10^−19^ *m*^2^/*V*^2^ (*SI*)) of other crystals found in the literature, e.g., bulk gold[85,86]. This demonstrates that FBHZ constitutes a promising object for future studies and applications in NLO and quantum dots[87]. The dynamic electrical properties of FBHZ in different solvent media are also reported. For example, the values of γ(-2ω;ω,ω,0) increased almost 62.65%, while for values β_||_(-2ω;ω,ω) and α(-ω,ω) the increases were 71.93% and 10.82%, respectively, compared to the solvent-free situation.

The results for ChelpG charges show that the weak hydrogen bond causes little charge transfer between C−H⋯F.

The *d*_*norm*_ surfaces (HS) corroborate previously discussed information about weak hydrogen bonds, but the C−H⋯F was an exception, as it was not clearly observed, even demonstrating d_*C12⋯F1*_ 3.477(6) Å and ∠_*C12−H12⋯F1*_ 141.45(3°. However, through the 2D fingerprint plot this contact rises to contribute 9.9%. The DSC/TGA and Hot-stage Microscopy analysis were performed, and the results show the crystal’s stability in relation to temperature variation.

We hope that the interesting dynamic physical properties displayed by this organic crystal can motivate researchers who are involved in modern photonic devices to explore its potential more thoroughly.

## Supporting information

S1 Fig Synthesis and characterization. (TIF)

S2 Fig Infrared spectrum of FBHZ (4000–400 cm-1). (TIF)

S3 Fig Mass spectrum of FBHZ. (TIF)

S4 Fig 1H NMR spectrum (1-dimensional) of FBHZ (DMSO-d6, 500 MHz) plotted as signal intensity vs. chemical shift (ppm) and the expanded region (range 7.0–12 ppm) (DMSO-d6, 500MHz). (TIF)

S5 Fig 13C NMR spectrum (1-dimensional) of FBHZ plotted as signal intensity vs. chemical shift (ppm) (DMSO-d6, 500 MHz). (TIF)

S6 Fig UV scan spectrum of FBHZ showing the baseline of ethanol 99% and the wavelength (λ) of maximum absorbance. (TIF)

S7 Fig The unit cell of FBHZ. (TIF)

S8 Fig Molecular orbital plots, showing HOMO-LUMO as obtained in the CAM-B3LYP/6-311+G(d) level of theory for Acetone solvent, for the FBHZ molecule. (TIF)

S9 Fig Molecular orbital plots, showing HOMO-LUMO as obtained in the CAM-B3LYP/6-311+G(d) level of theory for Chloroform solvent, for the FBHZ molecule. (TIF)

S10 Fig Molecular orbital plots, showing HOMO-LUMO as obtained in the CAM-B3LYP/6-311+G(d) level of theory for Dichloromethane solvent, for the FBHZ molecule. (TIF)

S11 Fig Molecular orbital plots, showing HOMO-LUMO as obtained in the CAM-B3LYP/6-311+G(d) level of theory for Ethanol solvent, for the FBHZ molecule. (TIF)

S12 Fig Molecular orbital plots, showing HOMO-LUMO as obtained in the CAM-B3LYP/6-311+G(d) level of theory for Methanol solvent, for the FBHZ molecule. (TIF)

S13 Fig Molecular orbital plots, showing HOMO-LUMO as obtained in the CAM-B3LYP/6-311+G(d) level of theory for Water solvent, for the FBHZ molecule. (TIF)

S1 Table Crystal data and structure refinement of FBHZ. (DOCX)

S2 Table Fractional atomic coordinates (×104) and equivalent isotropic displacement parameters (Å2×103) for FBHZ. Ueq is defined as 1/3 of of the trace of the orthogonalised UIJ tensor. (DOCX)

S3 Table Anisotropic displacement parameters (Å2×103) for FBHZ. The Anisotropic displacement factor exponent takes the form: -2π2[h2a*2U11+2hka*b*U12+…]. (DOCX)

S4 Table Bond lengths and angles between bonds for FBHZ. (DOCX)

S5 Table Hydrogen atom coordinates (Å×104) and isotropic displacement parameters (Å2×103) for FBHZ. (DOCX)

S6 Table CAM-B3LYP/6-311+G(d) results for the dynamic linear polarizability (10–24 esu), first hyperpolarizability (10–30 esu) and second hyperpolarizability (10–36 esu) of various solvent FBHZ for the frequency ω = 0.0428 a.u. (DOCX)
