# Supplementary figures and images for: Solid state characterization and theoretical study of non-linear optical properties of a Fluoro-N-Acylhydrazide derivative
Source: PLoS One. 2017 Apr 24;12(4):e0175859. doi: 10.1371/journal.pone.0175859 (PMC5402957; doi:10.1371/journal.pone.0175859)

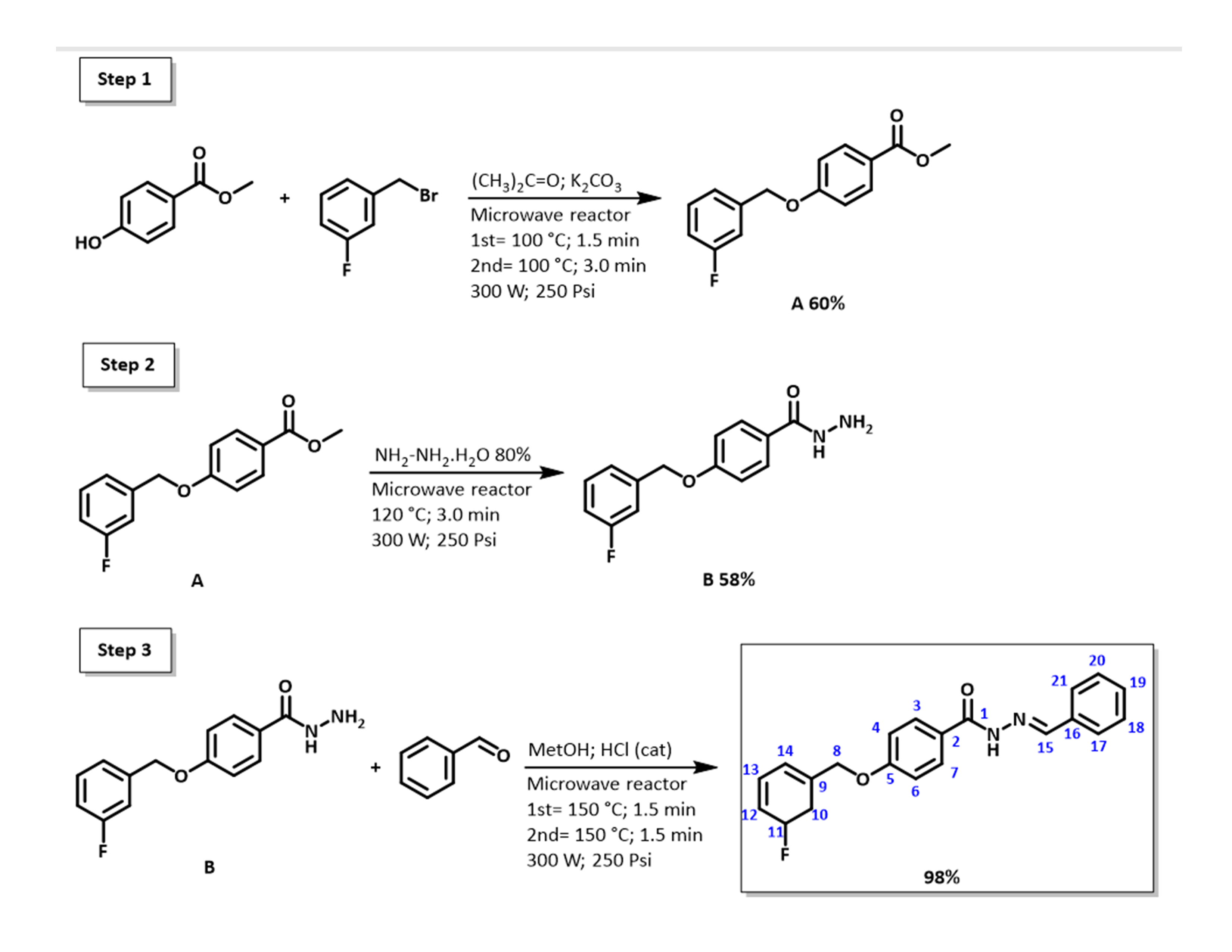

Supplement: S1 Fig — (TIF) [file pone.0175859.s001.tif]

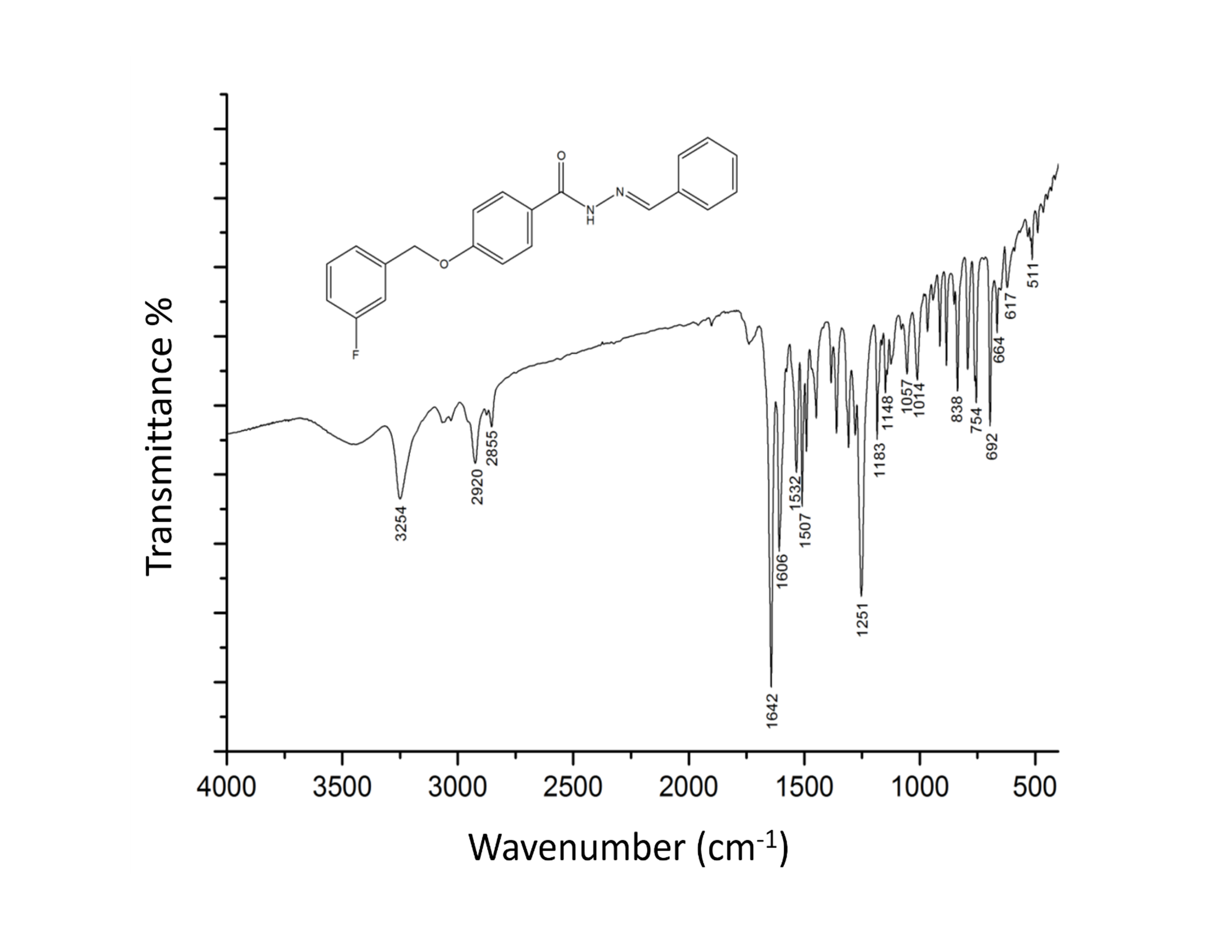

Supplement: S2 Fig — (TIF) [file pone.0175859.s002.tif]

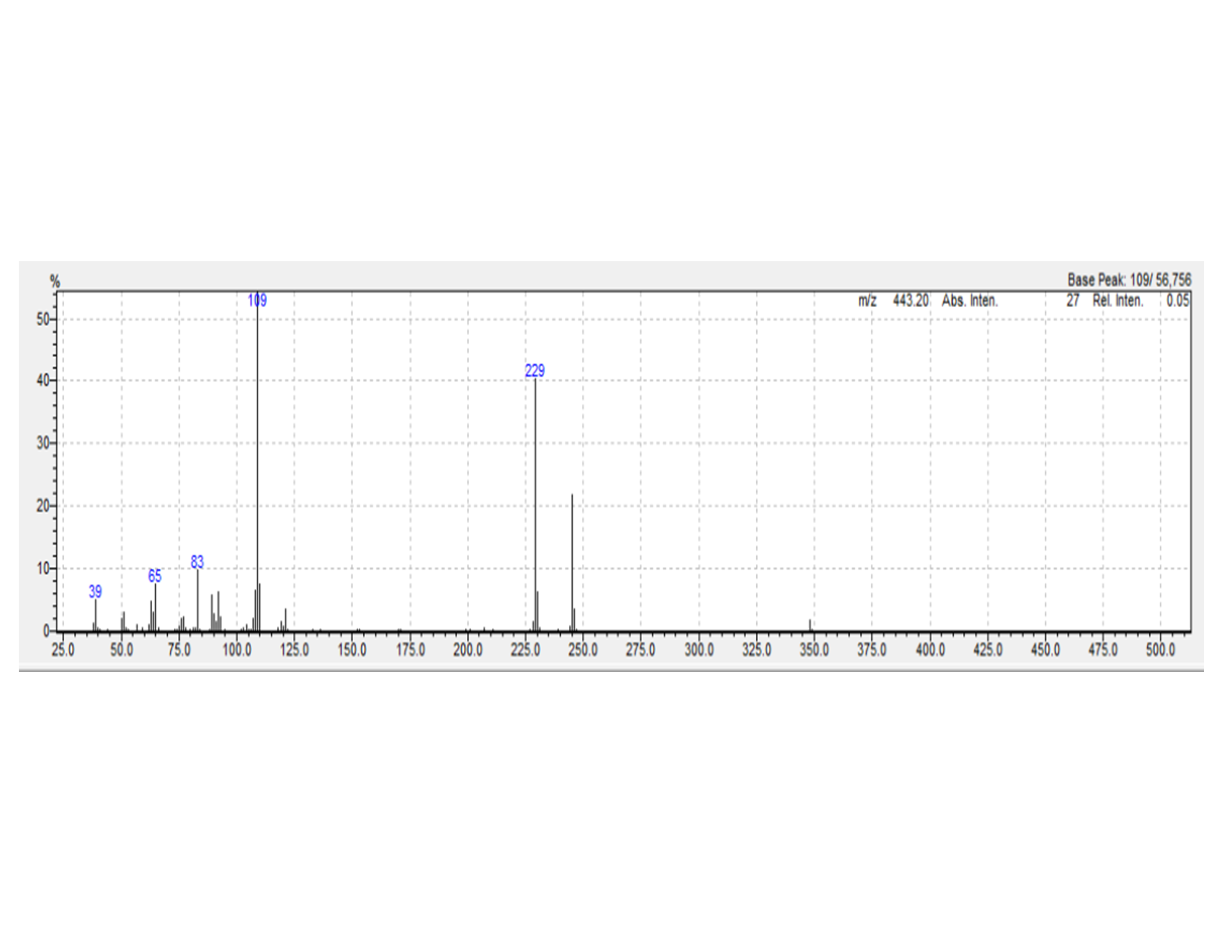

Supplement: S3 Fig — (TIF) [file pone.0175859.s003.tif]

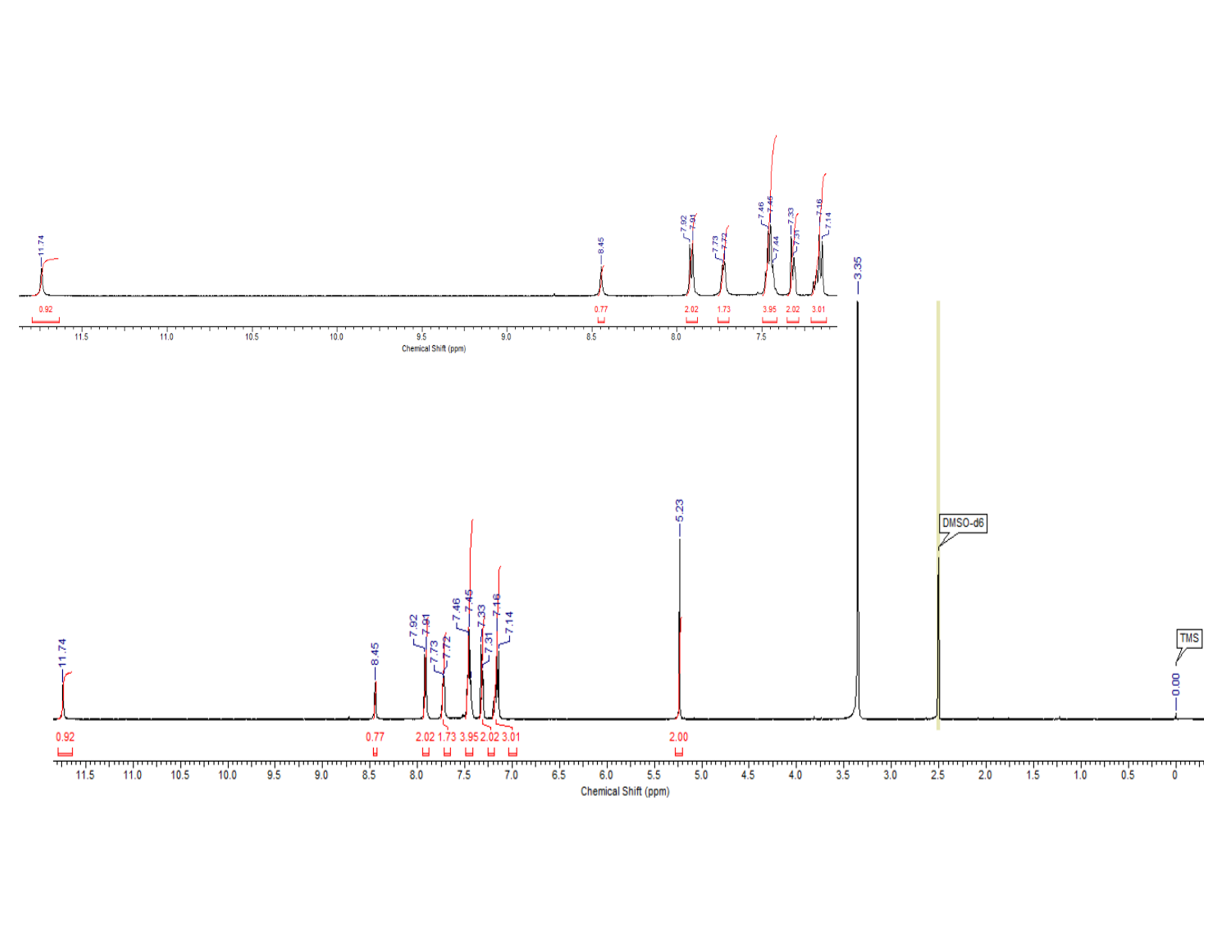

Supplement: S4 Fig — (TIF) [file pone.0175859.s004.tif]

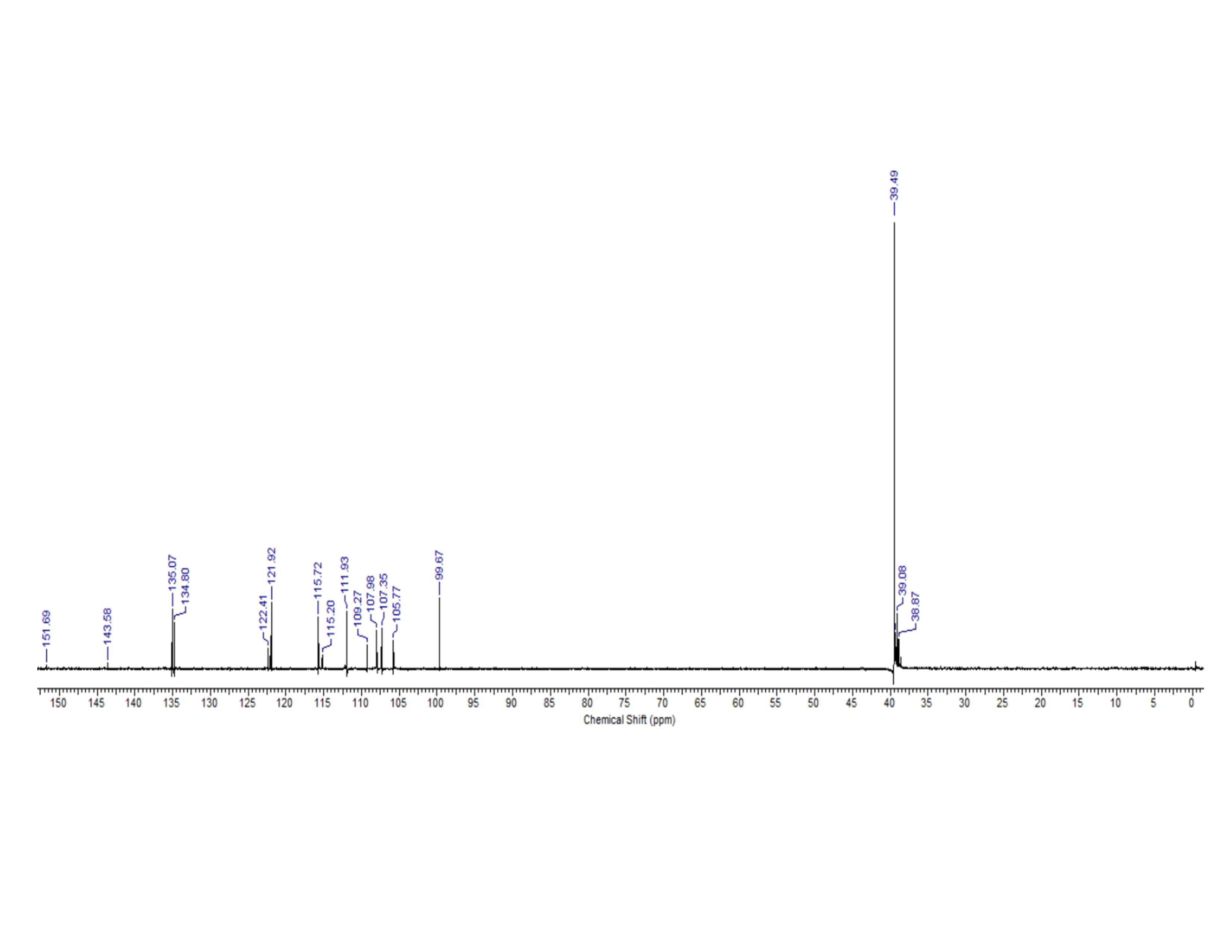

Supplement: S5 Fig — (TIF) [file pone.0175859.s005.tif]

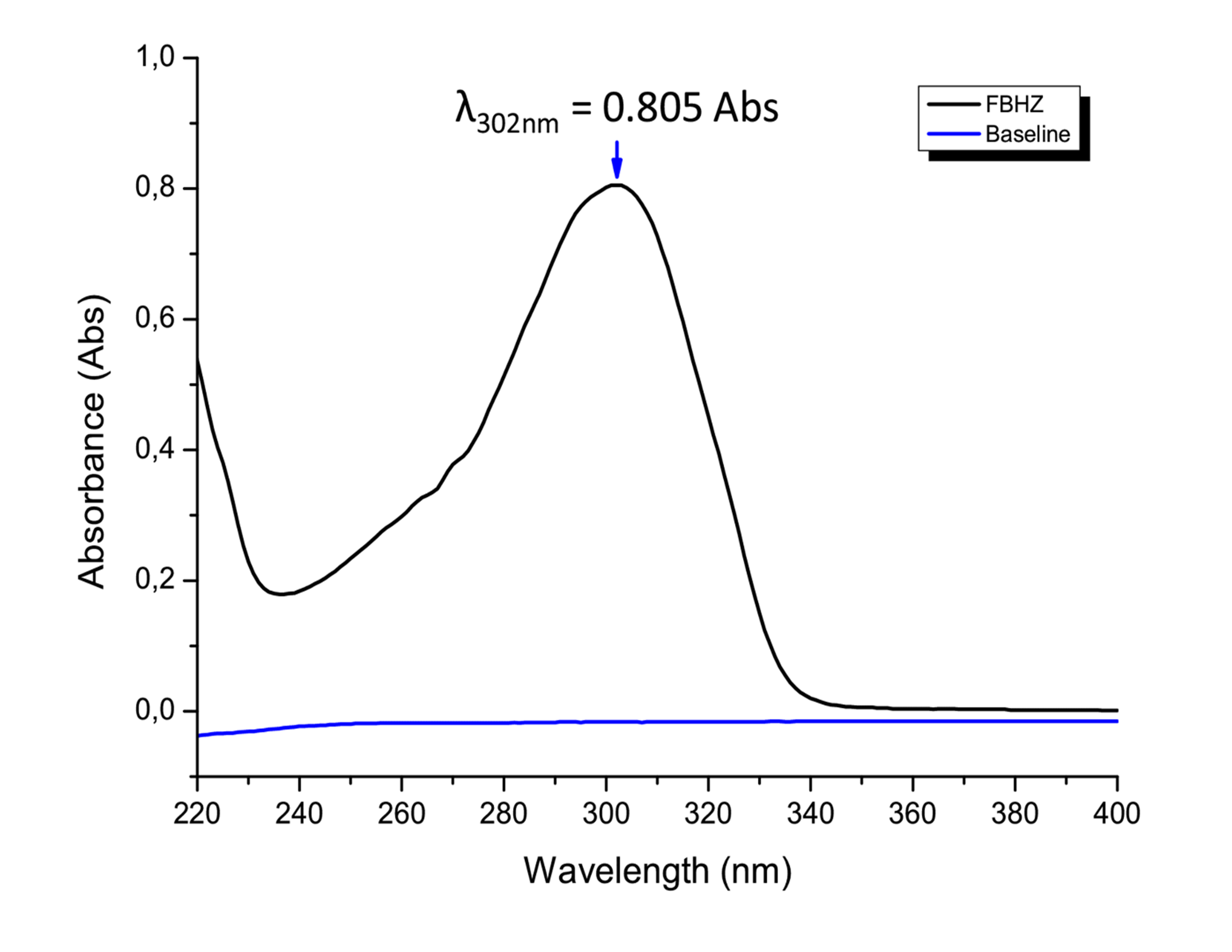

Supplement: S6 Fig — (TIF) [file pone.0175859.s006.tif]

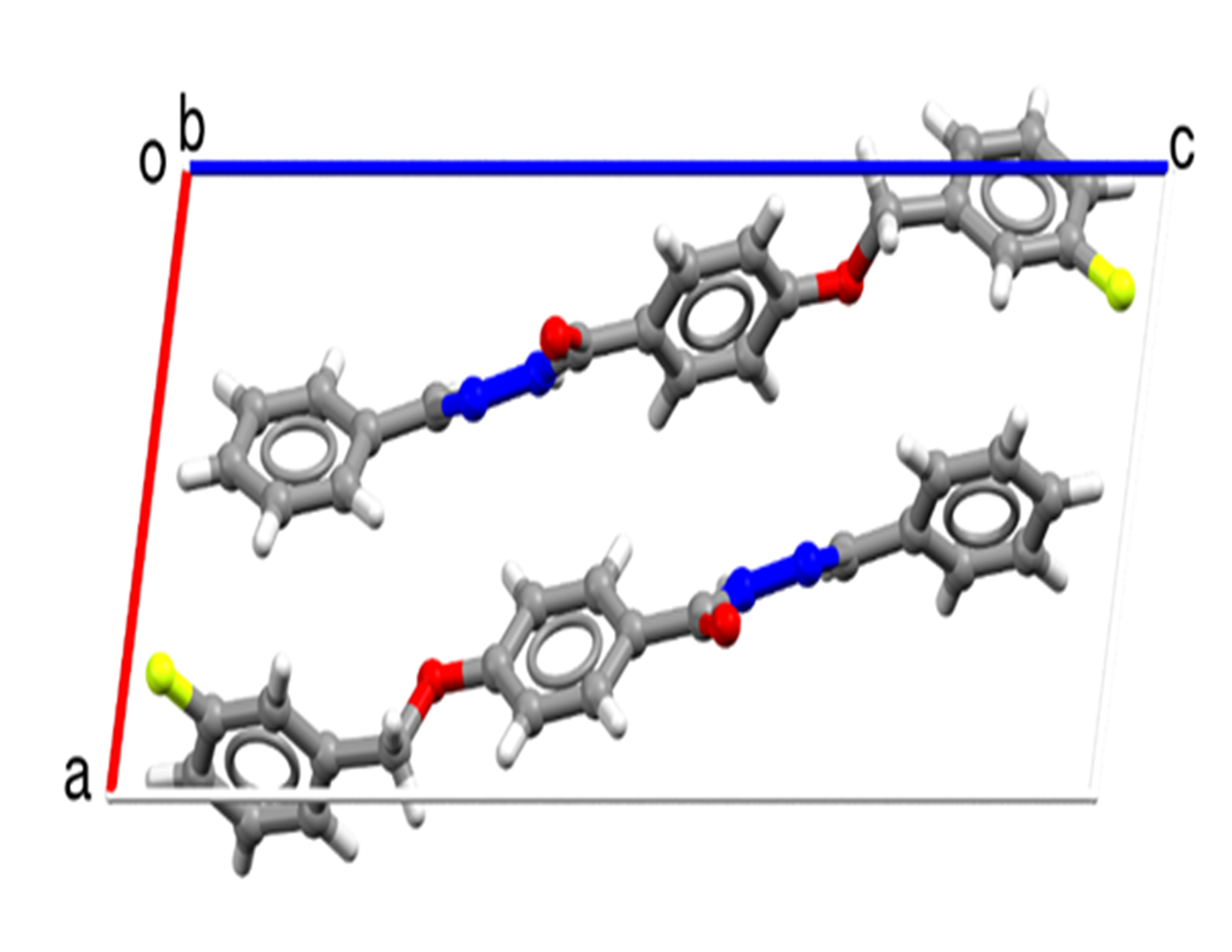

Supplement: S7 Fig — (TIF) [file pone.0175859.s007.tif]

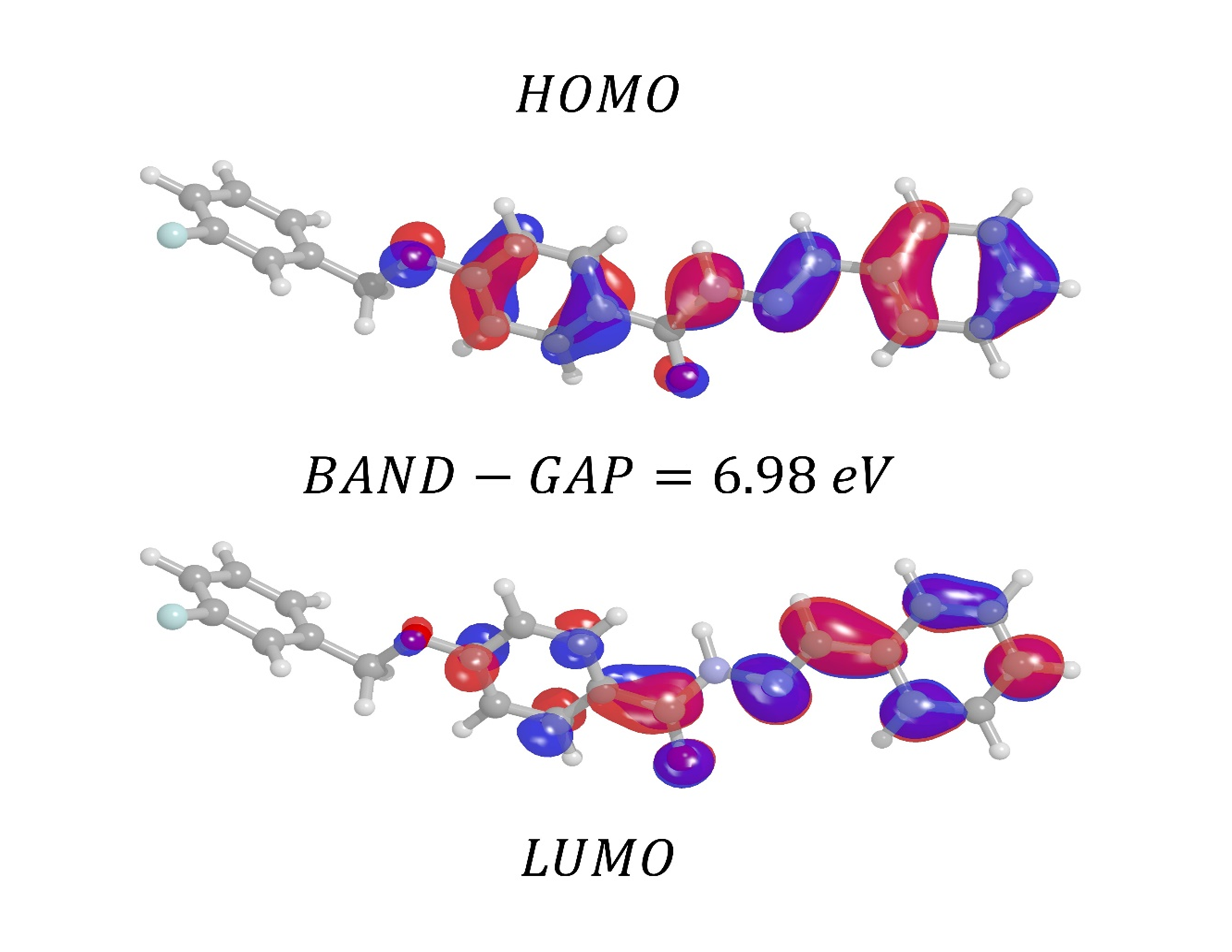

Supplement: S8 Fig — (TIF) [file pone.0175859.s008.tif]

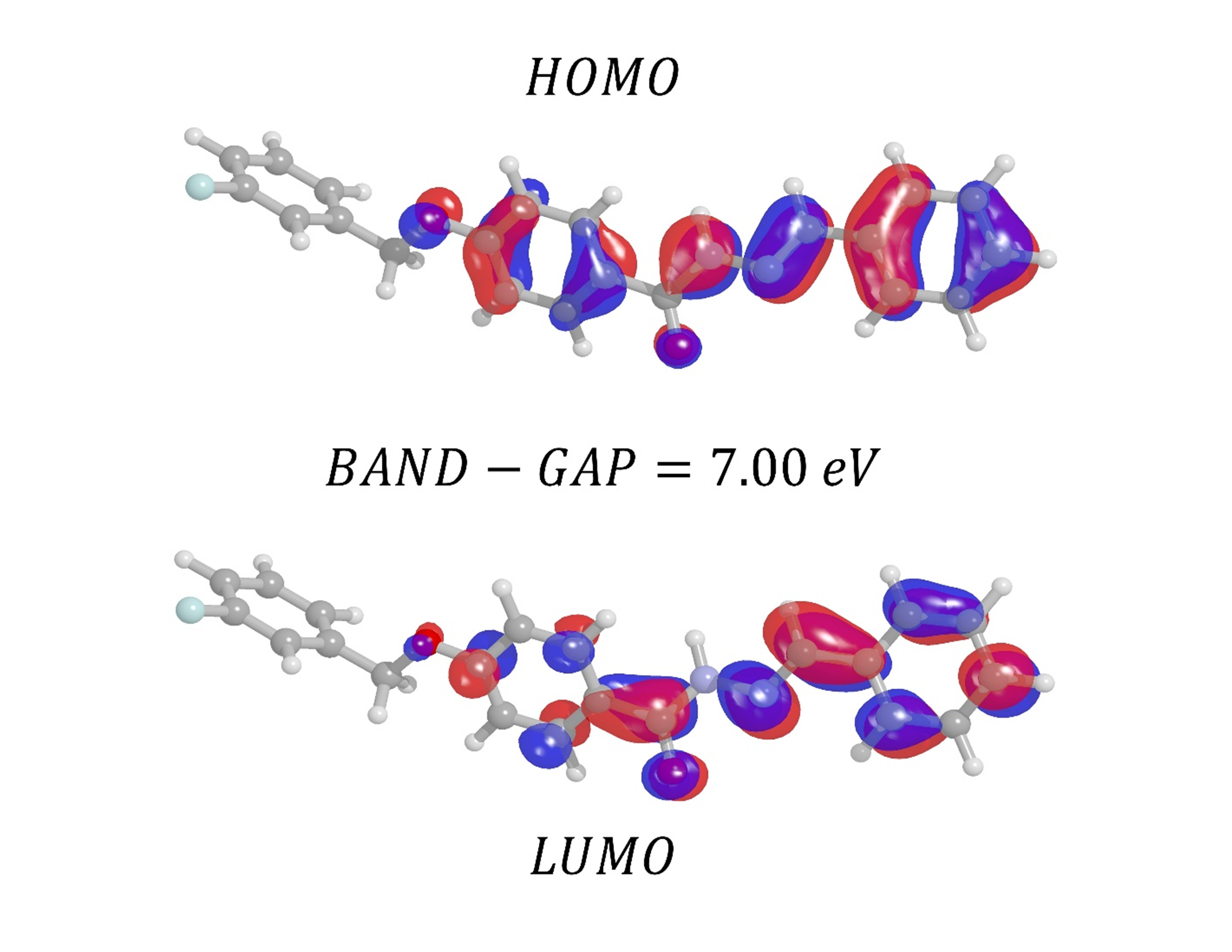

Supplement: S9 Fig — (TIF) [file pone.0175859.s009.tif]

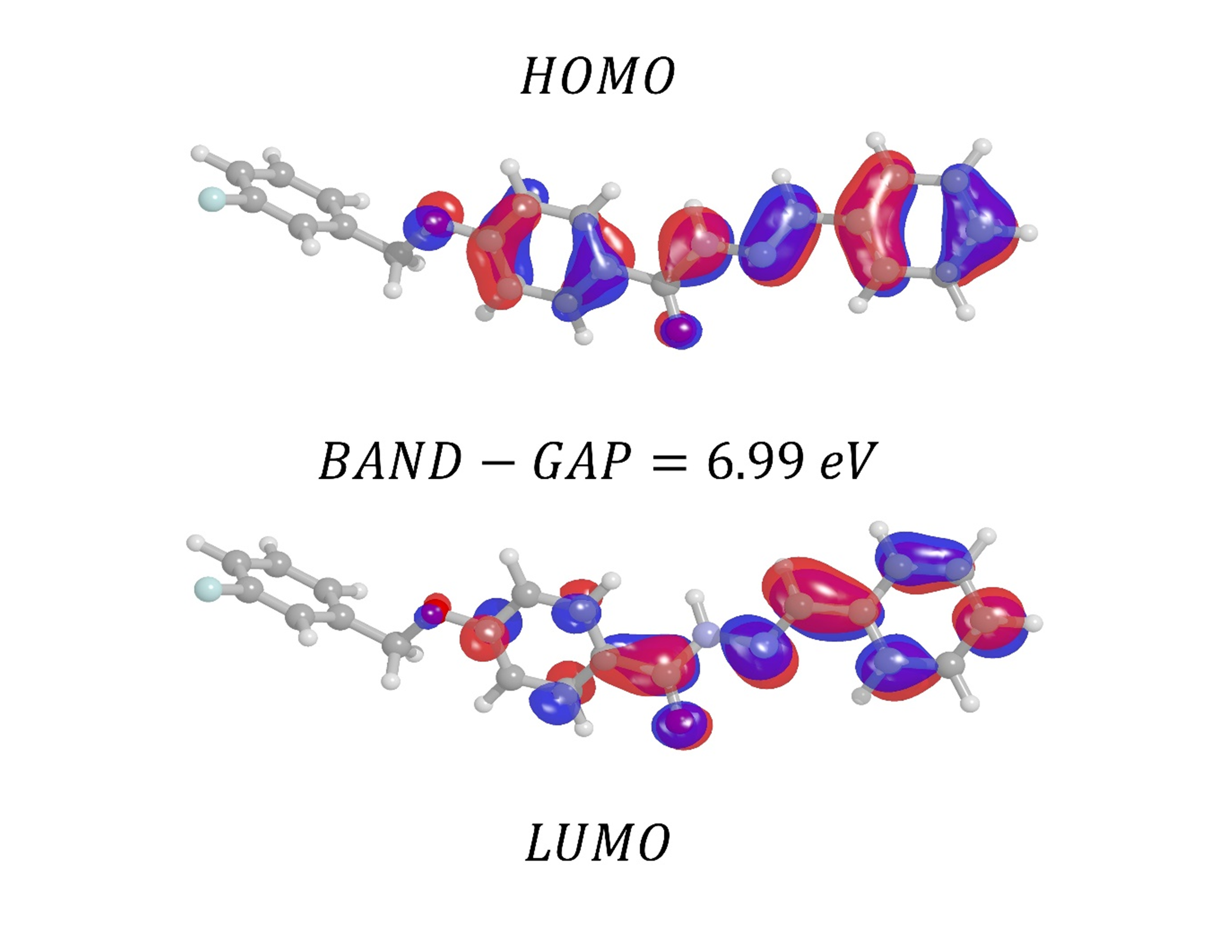

Supplement: S10 Fig — (TIF) [file pone.0175859.s010.tif]

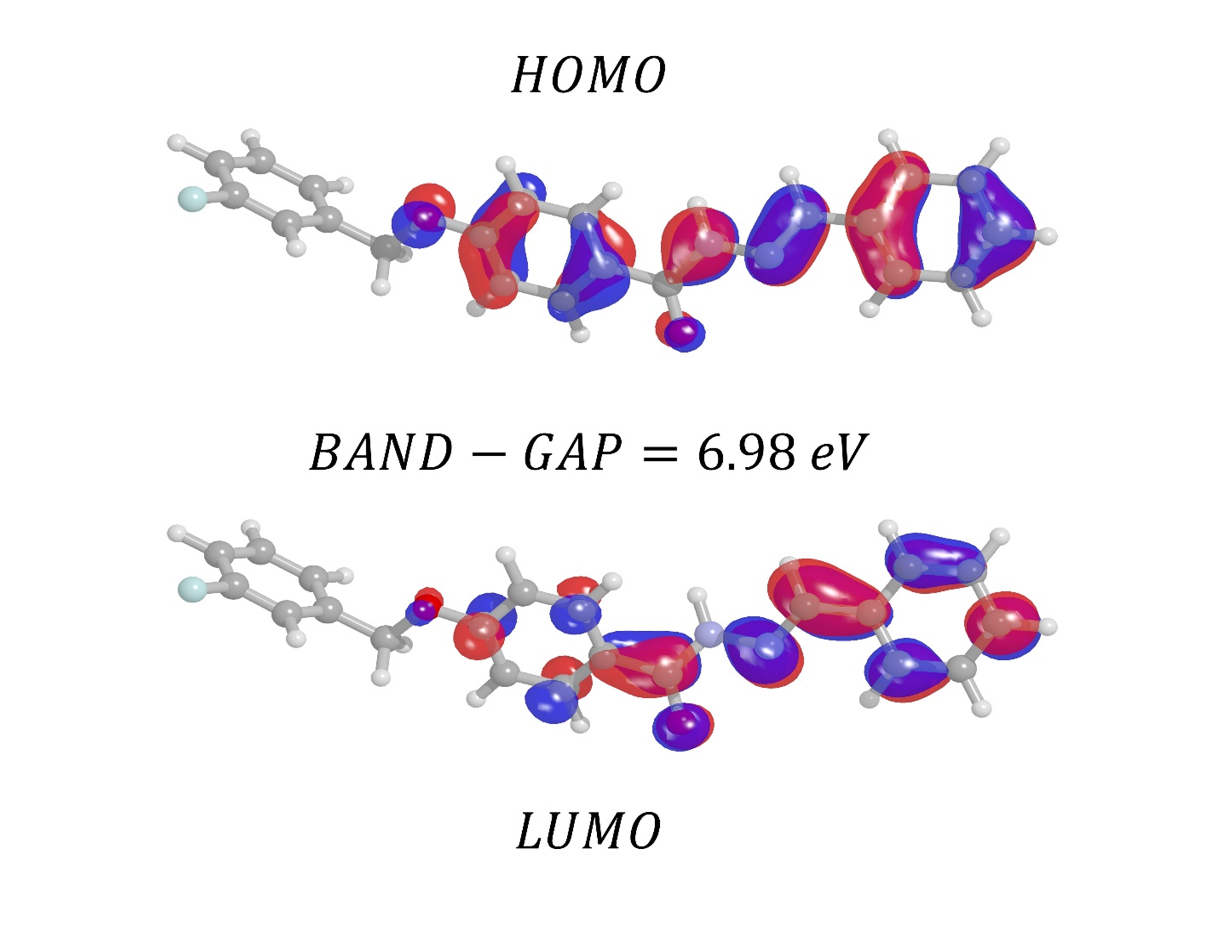

Supplement: S11 Fig — (TIF) [file pone.0175859.s011.tif]

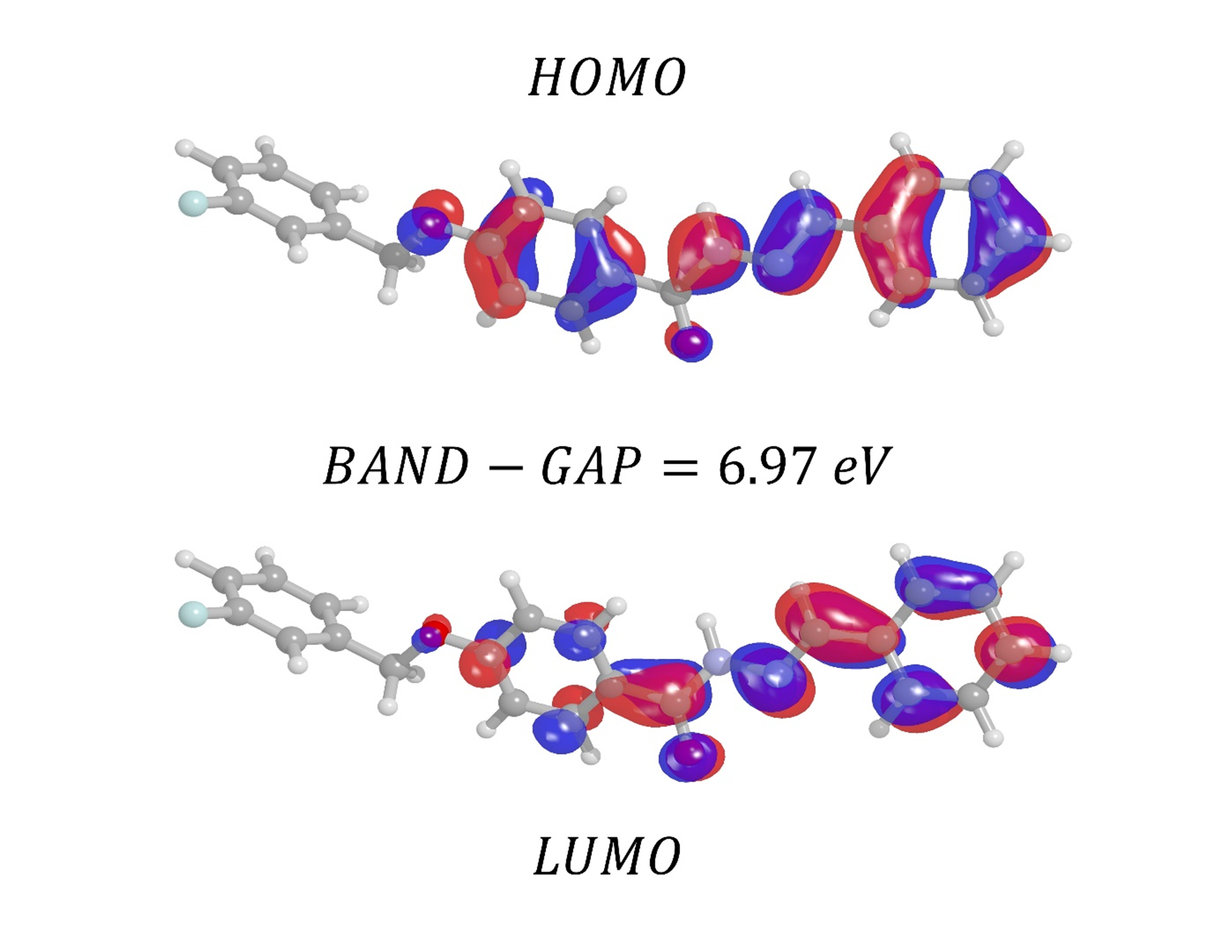

Supplement: S12 Fig — (TIF) [file pone.0175859.s012.tif]

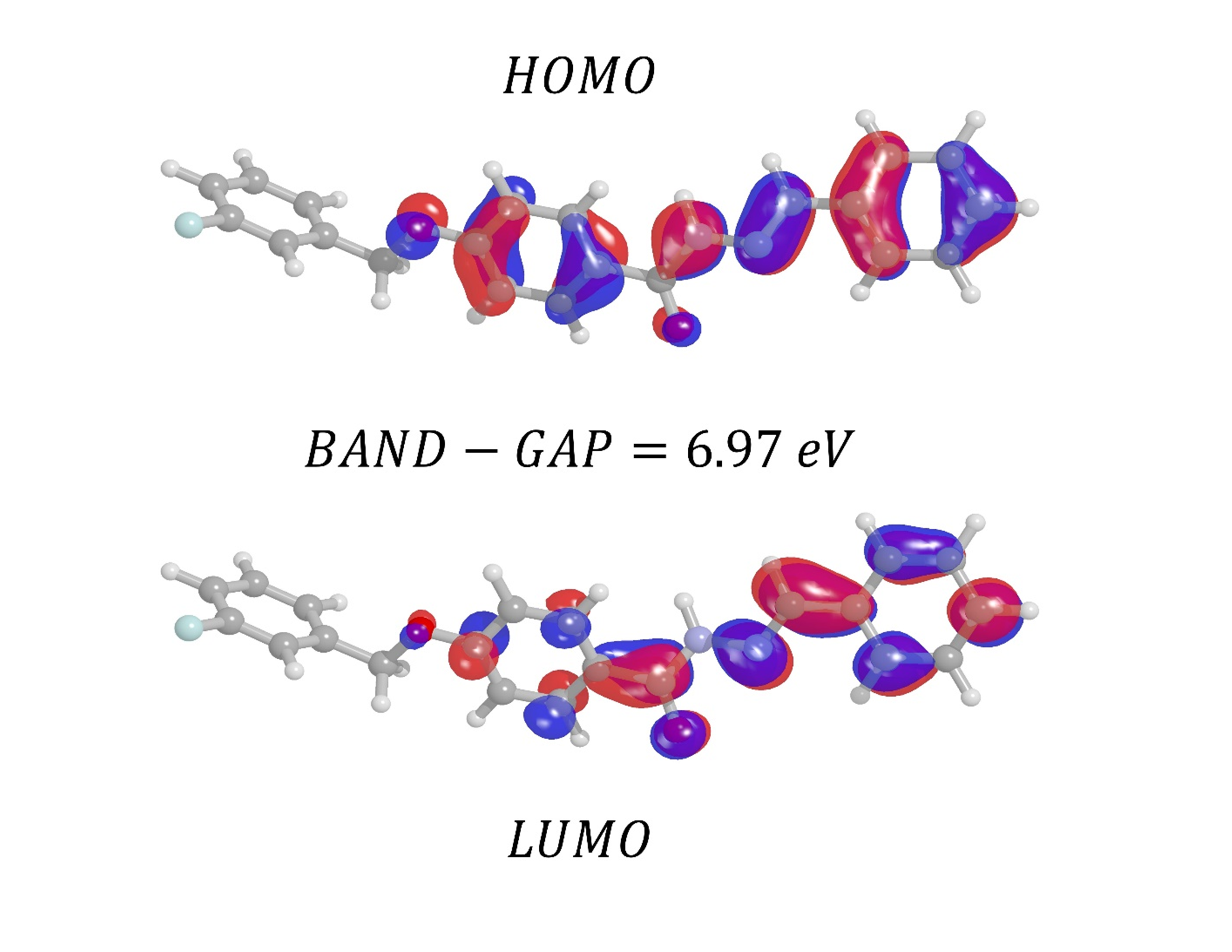

Supplement: S13 Fig — (TIF) [file pone.0175859.s013.tif]
