# Supplementary material for: Solid state characterization and theoretical study of non-linear optical properties of a Fluoro-N-Acylhydrazide derivative
Source: PLoS One. 2017 Apr 24;12(4):e0175859. doi: 10.1371/journal.pone.0175859 (PMC5402957; doi:10.1371/journal.pone.0175859)
Supplement: S1 Table — (DOCX) [file pone.0175859.s014.docx]

S1 Table. Crystal data and structure refinement of FBHZ.

| Identification code | CCDC 1497913 | |
| --- | --- | --- |
| Empirical formula | C_21_H_17_FN_2_O_2_ | |
| Formula weight | 348.36 | |
| Temperature/K | 296(2) | |
| Crystal system | Monoclinic | |
| Space group | P2_1_ | |
| Unit cell dimensions | 8.5766(5) Å | 90 ° |
|  | 5.1893(3) Å | 100.451(2) ° |
|  | 19.7867(10) Å | 90 ° |
| Volume/Å^3^ | 866.03(8) | |
| Z | 2 | |
| ρ_calc_g/cm^3^ | 1.336 | |
| μ/mm^‑1^ | 0.774 | |
| F(000) | 364.0 | |
| Crystal size/mm^3^ | 0 × 0 × 0 | |
| Radiation | CuKα (λ = 1.54184) | |
| 2Θ range for data collection/° | 9.09 to 136.902 | |
| Index ranges | -10 ≤ h ≤ 10, -6 ≤ k ≤ 5, -23 ≤ l ≤ 23 | |
| Reflections collected | 10407 | |
| Independent reflections | 3088 [R_int_ = 0.0436, R_sigma_ = 0.036] | |
| Data/restraints/parameters | 3088/1/236 | |
| Goodness-of-fit on F^2^ | 1.046 | |
| Final R indexes [I>=2σ (I)] | R_1_ = 0.0388, wR_2_ = 0.1071 | |
| Final R indexes [all data] | R_1_ = 0.0502, wR_2_ = 0.1117 | |
| Largest diff. peak/hole / e Å^-3^ | 0.18/-0.21 | |
| Flack parameter | 0.13(9) | |
