# Supplementary material for: Solid state characterization and theoretical study of non-linear optical properties of a Fluoro-N-Acylhydrazide derivative
Source: PLoS One. 2017 Apr 24;12(4):e0175859. doi: 10.1371/journal.pone.0175859 (PMC5402957; doi:10.1371/journal.pone.0175859)
Supplement: S2 Table — (DOCX) [file pone.0175859.s015.docx]

S2 Table. Fractional Atomic Coordinates (×10^4^) and Equivalent Isotropic Displacement Parameters (Å^2^×10^3^) for FBHZ. U_eq_ is defined as 1/3 of of the trace of the orthogonalised U_IJ_ tensor.

| **Atom** | ***x*** | ***y*** | ***z*** | **U(eq)** |
| --- | --- | --- | --- | --- |
| N2 | 6318(3) | 7701(4) | 6803.2(11) | 57.3(5) |
| O2 | 8179(3) | 8175(5) | 3132.1(9) | 70.6(6) |
| O1 | 7283(3) | 3916(4) | 6045.3(10) | 64.8(5) |
| N1 | 6727(3) | 8130(4) | 6169.6(11) | 56.7(5) |
| C5 | 7978(3) | 7565(6) | 3777.6(13) | 54.7(6) |
| C4 | 8685(4) | 5484(6) | 4158.5(14) | 63.5(7) |
| C3 | 8397(4) | 5076(6) | 4816.8(14) | 60.0(6) |
| C7 | 6723(3) | 8759(6) | 4718.4(13) | 59.7(6) |
| C1 | 7150(3) | 6106(5) | 5811.1(13) | 51.2(6) |
| C2 | 7429(3) | 6703(5) | 5106.6(12) | 49.2(6) |
| C6 | 6987(4) | 9167(6) | 4061.1(14) | 65.0(7) |
| C16 | 5771(4) | 9352(6) | 7848.8(15) | 63.7(7) |
| C15 | 6189(4) | 9678(6) | 7164.4(14) | 63.1(7) |
| C9 | 9375(3) | 7751(8) | 2147.1(15) | 69.3(8) |
| C19 | 5248(6) | 8633(9) | 9177(2) | 99.1(14) |
| C17 | 6354(5) | 11090(7) | 8362.2(18) | 84.1(10) |
| C8 | 9250(4) | 6623(8) | 2832.2(15) | 75.8(9) |
| C21 | 4891(5) | 7267(7) | 8003.4(19) | 81.1(10) |
| C10 | 8604(5) | 6585(10) | 1556.0(17) | 92.1(12) |
| C11 | 8782(5) | 7540(13) | 935.7(17) | 103.9(15) |
| C18 | 6109(6) | 10701(9) | 9027.0(19) | 96.4(13) |
| C14 | 10293(5) | 9856(10) | 2095(2) | 97.4(11) |
| C13 | 10433(6) | 10806(11) | 1454(3) | 119.0(17) |
| F1 | 8062(5) | 6300(12) | 357.2(13) | 191(2) |
| C20 | 4632(6) | 6965(8) | 8668(2) | 97.0(13) |
| C12 | 9672(6) | 9659(13) | 868(2) | 110.2(18) |
