# Supplementary material for: Solid state characterization and theoretical study of non-linear optical properties of a Fluoro-N-Acylhydrazide derivative
Source: PLoS One. 2017 Apr 24;12(4):e0175859. doi: 10.1371/journal.pone.0175859 (PMC5402957; doi:10.1371/journal.pone.0175859)
Supplement: S3 Table — (DOCX) [file pone.0175859.s016.docx]

S3 Table. Anisotropic Displacement Parameters (Å^2^×10^3^) for FBHZ. The Anisotropic displacement factor exponent takes the form: -2π^2^[h^2^a*^2^U_11_+2hka*b*U_12_+…].

| **Atom** | **U_11_** | **U_22_** | **U_33_** | **U_23_** | **U_13_** | **U_12_** |
| --- | --- | --- | --- | --- | --- | --- |
| N2 | 79.4(14) | 51.9(12) | 44.8(10) | 2.8(9) | 22.4(10) | -1(1) |
| O2 | 81.2(12) | 88.5(16) | 45.2(9) | 6.7(10) | 19.8(9) | 24.1(11) |
| O1 | 91.8(13) | 50.5(11) | 56.2(10) | 7.2(9) | 24.4(9) | 0.9(9) |
| N1 | 79.8(14) | 47.3(13) | 47.2(10) | 4.9(9) | 22.7(10) | -0.7(10) |
| C5 | 59.9(13) | 63.0(16) | 41.9(11) | 0.5(11) | 11.5(10) | 3.9(11) |
| C4 | 75.5(17) | 65.5(18) | 52.9(15) | -1.1(12) | 20.8(13) | 18.6(13) |
| C3 | 75.5(16) | 53.1(14) | 52.4(14) | 5.6(11) | 14.5(12) | 12.3(12) |
| C7 | 69.7(15) | 62.7(16) | 50.0(13) | 2.6(11) | 19.1(11) | 16.8(12) |
| C1 | 57.8(14) | 48.7(14) | 48.5(13) | 1.4(11) | 13.5(10) | -2.9(10) |
| C2 | 56.3(13) | 48.1(14) | 44.0(12) | -2.3(10) | 11.2(10) | -2.4(10) |
| C6 | 77.6(17) | 70.1(19) | 48.8(13) | 10.0(12) | 15.0(12) | 21.4(14) |
| C16 | 88.8(18) | 52.3(16) | 56.1(15) | 3.9(12) | 29.6(13) | 10.4(13) |
| C15 | 89.0(18) | 50.4(16) | 55.2(14) | 6.2(12) | 27.3(13) | 1.5(13) |
| C9 | 62.7(15) | 97(2) | 51.0(14) | 1.5(15) | 17.1(12) | 18.3(16) |
| C19 | 150(4) | 93(3) | 69(2) | 21(2) | 60(2) | 39(3) |
| C17 | 128(3) | 65.2(19) | 66.4(19) | -6.5(16) | 38.1(19) | -1.0(19) |
| C8 | 83(2) | 98(3) | 50.3(15) | 4.6(15) | 23.5(14) | 24.9(17) |
| C21 | 112(3) | 63(2) | 79(2) | 2.4(16) | 43.9(19) | -2.1(17) |
| C10 | 84(2) | 138(4) | 55.3(18) | -1(2) | 16.2(15) | -8(2) |
| C11 | 104(3) | 162(4) | 47.9(17) | 10(2) | 19.7(17) | 22(3) |
| C18 | 147(4) | 88(3) | 61.1(19) | -6.4(18) | 38(2) | 13(2) |
| C14 | 96(3) | 107(3) | 89(3) | 6(2) | 16(2) | -6(2) |
| C13 | 109(3) | 117(4) | 141(5) | 38(4) | 50(3) | 2(3) |
| F1 | 204(4) | 306(6) | 54.6(14) | -13(2) | 0.9(17) | -17(4) |
| C20 | 146(4) | 72(2) | 91(3) | 16(2) | 69(3) | 7(2) |
| C12 | 101(3) | 155(5) | 86(3) | 50(3) | 47(2) | 49(3) |
