# Supplementary material for: Solid state characterization and theoretical study of non-linear optical properties of a Fluoro-N-Acylhydrazide derivative
Source: PLoS One. 2017 Apr 24;12(4):e0175859. doi: 10.1371/journal.pone.0175859 (PMC5402957; doi:10.1371/journal.pone.0175859)
Supplement: S4 Table — (DOCX) [file pone.0175859.s017.docx]

S4 Table. Bond Lengths and Angles between bonds for FBHZ.

| **Atoms** | **Length (Å)** | **Atoms** | **Angles (°)** | **Atoms** | **Angles (°)** |
| --- | --- | --- | --- | --- | --- |
| N2−C15 | 1.267(4) | C15−N2−N1 | 116.4(2) | C10−C11−C12 | 122.8(5) |
| N2−N1 | 1.380(3) | C5−O2−C8 | 117.4(2) | C10−C11−F1 | 118.7(6) |
| O2−C5 | 1.357(3) | C1−N1−N2 | 119.2(2) | C12−C11−F1 | 118.5(4) |
| O2−C8 | 1.429(3) | O2−C5−C6 | 115.8(2) | C19−C18−C17 | 120.1(4) |
| O1−C1 | 1.225(3) | O2−C5−C4 | 125.0(2) | C9−C14−C13 | 120.2(5) |
| N1−C1 | 1.354(3) | C6−C5−C4 | 119.2(2) | C12−C13−C14 | 121.1(5) |
| C5−C6 | 1.379(4) | C3−C4−C5 | 119.6(2) | C19−C20−C21 | 121.6(4) |
| C5−C4 | 1.392(4) | C2−C3−C4 | 121.4(3) | C11−C12−C13 | 117.4(4) |
| C4−C3 | 1.385(4) | C6−C7−C2 | 120.7(2) |  |  |
| C3−C2 | 1.380(4) | O1−C1−N1 | 122.5(2) |  |  |
| C7−C6 | 1.376(3) | O1−C1−C2 | 121.8(2) |  |  |
| C7−C2 | 1.388(4) | N1−C1−C2 | 115.7(2) |  |  |
| C1−C2 | 1.489(3) | C3−C2−C7 | 118.4(2) |  |  |
| C16−C17 | 1.382(5) | C3−C2−C1 | 118.2(2) |  |  |
| C16−C21 | 1.385(5) | C7−C2−C1 | 123.3(2) |  |  |
| C16−C15 | 1.472(3) | C7−C6−C5 | 120.7(3) |  |  |
| C9−C14 | 1.361(6) | C17−C16−C21 | 119.1(3) |  |  |
| C9−C10 | 1.375(5) | C17−C16−C15 | 119.0(3) |  |  |
| C9−C8 | 1.498(4) | C21−C16−C15 | 121.8(3) |  |  |
| C19−C20 | 1.359(7) | N2−C15−C16 | 119.1(3) |  |  |
| C19−C18 | 1.365(7) | C14−C9−C10 | 118.9(4) |  |  |
| C17−C18 | 1.384(5) | C14−C9−C8 | 121.3(4) |  |  |
| C21−C20 | 1.383(5) | C10−C9−C8 | 119.7(4) |  |  |
| C10−C11 | 1.358(5) | C20−C19−C18 | 119.6(3) |  |  |
| C11−C12 | 1.359(8) | C16−C17−C18 | 120.4(4) |  |  |
| C11−F1 | 1.360(6) | O2−C8−C9 | 108.2(3) |  |  |
| C14−C13 | 1.386(6) | C20−C21−C16 | 119.2(4) |  |  |
| C13−C12 | 1.361(8) | C11−C10−C9 | 119.5(5) |  |  |
