# Supplementary material for: Solid state characterization and theoretical study of non-linear optical properties of a Fluoro-N-Acylhydrazide derivative
Source: PLoS One. 2017 Apr 24;12(4):e0175859. doi: 10.1371/journal.pone.0175859 (PMC5402957; doi:10.1371/journal.pone.0175859)
Supplement: S5 Table — (DOCX) [file pone.0175859.s018.docx]

S5 Table. Hydrogen Atom Coordinates (Å×10^4^) and Isotropic Displacement Parameters (Å^2^×10^3^) for FBHZ.

| **Atom** | ***x*** | ***y*** | ***z*** | **U(eq)** |
| --- | --- | --- | --- | --- |
| H1 | 6712 | 9665 | 6004 | 68 |
| H4 | 9347 | 4375 | 3972 | 76 |
| H3 | 8866 | 3676 | 5069 | 72 |
| H7 | 6064 | 9874 | 4904 | 72 |
| H6 | 6491 | 10538 | 3805 | 78 |
| H15 | 6356 | 11314 | 7000 | 76 |
| H19 | 5084 | 8368 | 9623 | 119 |
| H17 | 6914 | 12529 | 8260 | 101 |
| H8A | 8861 | 4867 | 2776 | 91 |
| H8B | 10284 | 6600 | 3128 | 91 |
| H21 | 4479 | 6086 | 7664 | 97 |
| H10 | 7965 | 5153 | 1581 | 110 |
| H18 | 6532 | 11851 | 9373 | 116 |
| H14 | 10827 | 10658 | 2490 | 117 |
| H13 | 11058 | 12252 | 1424 | 143 |
| H20 | 4021 | 5587 | 8770 | 116 |
| H12 | 9757 | 10299 | 437 | 132 |
