# Supplementary material for: Solid state characterization and theoretical study of non-linear optical properties of a Fluoro-N-Acylhydrazide derivative
Source: PLoS One. 2017 Apr 24;12(4):e0175859. doi: 10.1371/journal.pone.0175859 (PMC5402957; doi:10.1371/journal.pone.0175859)
Supplement: S6 Table — (DOCX) [file pone.0175859.s019.docx]

S6 Table. CAM-B3LYP/6-311+G(d) results for the dynamic linear polarizability (10^-24^ esu), first hyperpolarizability (10^-30^ esu) and second hyperpolarizability (10^-36^ esu) of various solvent FBHZ for the frequency ω=0.0428 a.u.

| **Medium** | **α(-ω;ω)** | **β\|\|(-ω;ω,0)** | **β\|\|(-2ω;ω,ω)** | **γ(-ω;ω,0,0)** | **γ(-2ω;ω,ω,0)** |
| --- | --- | --- | --- | --- | --- |
| Gas-Phase | 43.62 | 7.24 | 9.19 | 96.76 | 129.38 |
| Methanol | 47.58 | 11.81 | 14.37 | 162.26 | 202.29 |
| Water | 47.63 | 11.96 | 14.55 | 164.40 | 204.32 |
| Acetona | 47.84 | 11.83 | 14.46 | 161.69 | 203.27 |
| Ethanol | 47.86 | 11.88 | 14.51 | 163.68 | 204.22 |
| DiMethylSulfoxide | 48.34 | 12.81 | 15.79 | 166.98 | 210.43 |
| Dichloromethane | 43.36 | 11.63 | 14.37 | 158.14 | 203.16 |
| Chloroform | 48.48 | 11.21 | 13.99 | 150.47 | 197.40 |
